# The pan-genome of *Aspergillus fumigatus* provides a high-resolution view of its population structure revealing high levels of lineage-specific diversity driven by recombination

**DOI:** 10.1371/journal.pbio.3001890

**Published:** 2022-11-17

**Authors:** Lotus A. Lofgren, Brandon S. Ross, Robert A. Cramer, Jason E. Stajich

**Affiliations:** 1 Department of Microbiology and Plant Pathology, University of California Riverside, Riverside, California, United States of America; 2 Department of Biology, Duke University, Durham, North Carolina, United States of America; 3 Dartmouth Geisel School of Medicine in the Department of Microbiology and Immunology, Dartmouth, Hanover, New Hampshire, United States of America; University of California San Francisco, UNITED STATES

## Abstract

*Aspergillus fumigatus* is a deadly agent of human fungal disease where virulence heterogeneity is thought to be at least partially structured by genetic variation between strains. While population genomic analyses based on reference genome alignments offer valuable insights into how gene variants are distributed across populations, these approaches fail to capture intraspecific variation in genes absent from the reference genome. Pan-genomic analyses based on de novo assemblies offer a promising alternative to reference-based genomics with the potential to address the full genetic repertoire of a species. Here, we evaluate 260 genome sequences of *A*. *fumigatus* including 62 newly sequenced strains, using a combination of population genomics, phylogenomics, and pan-genomics. Our results offer a high-resolution assessment of population structure and recombination frequency, phylogenetically structured gene presence–absence variation, evidence for metabolic specificity, and the distribution of putative antifungal resistance genes. Although *A*. *fumigatus* disperses primarily via asexual conidia, we identified extraordinarily high levels of recombination with the lowest linkage disequilibrium decay value reported for any fungal species to date. We provide evidence for 3 primary populations of *A*. *fumigatus*, with recombination occurring only rarely between populations and often within them. These 3 populations are structured by both gene variation and distinct patterns of gene presence–absence with unique suites of accessory genes present exclusively in each clade. Accessory genes displayed functional enrichment for nitrogen and carbohydrate metabolism suggesting that populations may be stratified by environmental niche specialization. Similarly, the distribution of antifungal resistance genes and resistance alleles were often structured by phylogeny. Altogether, the pan-genome of *A*. *fumigatus* represents one of the largest fungal pan-genomes reported to date including many genes unrepresented in the Af293 reference genome. These results highlight the inadequacy of relying on a single-reference genome-based approach for evaluating intraspecific variation and the power of combined genomic approaches to elucidate population structure, genetic diversity, and putative ecological drivers of clinically relevant fungi.

## Abstract 2

A combined population genomics, phylogenomics, and pan-genomics approach reveals evidence for high levels of recombination and metabolic diversity in the opportunistic fungal pathogen Aspergillus fumigatus.

## Introduction

*Aspergillus fumigatus* is one of the most common etiological agents of human fungal disease [1,2]. The spectrum of diseases attributed to *A*. *fumigatus* is remarkable. In immunocompromised patients, invasive *A*. *fumigatus* infection causes up to 90% mortality, even with aggressive treatment [3], an outcome that is further complicated by the increasing presence of triazole-resistant strains [4]. Phenotypic heterogeneity in growth and virulence is well documented in *A*. *fumigatus* [5–9] and thought to be partially structured by genetic variation between strains [10]. These intraspecific genetic differences likely represent both gene variants (insertions/deletions and single nucleotide polymorphisms) [11] and differences in gene presence–absence, copy number, and structural arrangements [12,13]. While population-genomic analyses based on reference genome alignment have provided valuable insights into how gene variants are distributed across populations, these approaches fail to capture intraspecific variation in genomic regions absent from the reference genome. In contrast to reference-based genomics, pan-genomic analyses based on de novo assemblies offer the potential to address the full genetic repertoire of a species. For *A*. *fumigatus* pathogenesis and virulence, identification of strain-specific genes and alleles is expected to further clarify the role of fungal genetic variation in disease outcomes.

The concept of a pan-genome, here defined as all genetic elements present across a species [14], recognizes that while many genes are fixed within a population and present in all individuals (core genes), others display substantial presence–absence variation (dispensable or accessory genes). Core genes are expected to be enriched in housekeeping functions and clustered in protected areas of chromosomes, where they are conserved by stronger efficiency of purifying selection [15]. Conversely, accessory genes are more likely to encode lineage-specific proteins that facilitate environmental adaptation [16], such as genes involved in secondary metabolism and niche specificity and tend to be concentrated in highly variable, rapidly evolving subtelomeric regions and adjacent to transposable elements (TEs) [17–20]. Variation in the accessory genome is essential to facilitating diversifying selection and adaptation to population-specific environmental pressures and may be particularly important for fungal pathogen adaptation [15,21].

In addition to its importance as a pathogen of humans and other animals [22–24], *A*. *fumigatus* is an ubiquitous plant litter saprophyte and plays a substantial ecological role in carbon and nutrient cycling, enabled by the wide range of carbohydrate-active enzymes encoded in the genome and involved in the decay of organic matter [2,25]. The fungus primarily reproduces asexually, via the production of prolific, stress-resistant, hydrophobic conidia [26]. This combination of traits contribute to the high dispersibility of the species, where conidia are rendered airborne by the slightest wind currents or easily carried to new locations by water, swarming soil bacteria, and soil invertebrates [27]. Due to this exceptional dispersibility and the assumption of extreme substrate generalism, *A*. *fumigatus* was originally thought to represent a single homogenous population [28,29]. However, recent investigations into *A*. *fumigatus* population structure have found mixed evidence for population stratification with results heavily dependent on the methods used for analysis and the number of isolates under consideration [30–33]. Interestingly, previous studies have shown little to no correlation of population structure with geography [32,34], as genetically identical (clonal) isolates collected from disparate locations across the globe [32]. This lack of geographic structure would seem to support a model of panmixia, but geography is not the only potential force structuring fungal populations, which must adapt to a plethora of environmental stressors and niche opportunities. However, niche specificity and the potential for environmental drivers to structure population stratification in *A*. *fumigatus* have yet to be investigated. Given its primary ecological strategy as a plant litter saprophyte, we hypothesized that population structure in *A*. *fumigatus* would be underpinned by metabolic specificity, with population-specific genomic variation concentrated in genes relevant to nutrient mining. Such metabolic variation may have evolved to access different plant substrates, but have important implications for human and animal disease [35].

Because *A*. *fumigatus* is thought to reproduce primarily clonally (and rarely sexually) [36], population structure is likely to be complicated by local clonal population bursts followed by dispersal. One well-studied example of this is the movement of *cyp51A* resistance alleles across the world. The *cyp51A* gene encodes 14-alpha sterol demethylase and is the primary drug target for azole antifungals [37]. Environmental pressures for allelic variation at this critical azole target are hypothesized to come primarily from the widespread use of agricultural antifungals [38]. Human-to-human transmission was originally thought to represent a dead-end for the species; however, recent evidence support occasional hospital-related transmission [39,40] and the possibility for clinical antifungal treatment to represent a secondary source of antifungal pressure. Although the *cyp51A* mutation has been found in multiple genetic backgrounds [30], the distribution of drug-resistant isolates across the phylogeny is nonrandom, with resistant strains showing low levels of genetic diversity and close genetic relatedness, likely representing high levels of clonal reproduction and a selective sweep for resistant phenotypes [32,34]. Although *cyp51A* mutations are the best studied drug resistance mechanism in *A*. *fumigatus*, mutations in *cyp51A* only account for an estimated 43% of resistant isolates [41]. While many other genes have been implicated in azole resistance [42], it is unknown whether mutations in these genes are similarly structured by phylogeny.

A sexual cycle has been documented in *A*. *fumigatus* [36]. However, the frequency of recombination events and their capacity to shape *A*. *fumigatus* populations is unknown. Whereas widely distributed generalist species with large effective population sizes are assumed to have larger pan-genomes, frequent clonal reproduction is thought to limit pan-genome size [21,43]. Generally, the evolution of large pan-genomes implies frequent gene flow, which may take the form of sexual or pseudo-sexual exchange, or significant horizontal gene transfer [44]. In open pan-genomes, the ratio of core/accessory genes is expected to decrease with an increasing number of genomes analyzed. Conversely, closed pan-genomes will quickly reach a saturation point at which adding new genomes to the analysis adds few new genes to the total pool. It is unclear how a species like *A*. *fumigatus*, which demonstrates ubiquitous geographic distribution, assumed substrate generalism, primary clonal reproduction, but potentially high recombination rates [45], fits into these expectations.

## Methods

### DNA preparation

All strains sequenced in this project were isolated on *Aspergillus* minimal medium [46] by picking single germinated spores after 16 h of growth at 30°C. Cultures were grown for DNA extraction in liquid minimal media with 1% (w/v) D-Glucose, 0.5% yeast extract (w/v, Beckton-Dickinson), 20 ml 50× salt solution, 1 ml trace elements solution, 20 mM NaNO_3_, pH adjusted to 6.5 using NaOH, and autoclaved for 20 min at 121°C.

### Genome sequencing

After 24 h of growth, cultures were lyophilized for approximately 10 h and homogenized using a bead beater (1 min with 2.3-mm beads). DNA was extracted using a LETS buffer protocol [47] modified with the addition of a 1-h RNAse treatment prior to phenol–chloroform extraction. DNA concentration was quantified using a Qubit 2.0 Fluorometer (Invitrogen) with the Broad Range protocol. Genomic sequencing was carried out on either Illumina NovaSeq 6000 or NextSeq 500 machines. DNA sequencing libraries were prepared using either the NEBNext Ultra II DNA Library Prep Kit (for NovaSeq 6000 sequenced genomes) or the SeqOnce DNA library kit utilizing Covaris mechanized shearing (for the NextSeq 500 sequenced isolates), both following manufacturer recommendations with paired end library construction and barcoding for multiplexing. All genome sequencing data generated for this project was deposited into the NCBI Sequence Read Archive under BioProject no. PRJNA666940.

### Strain selection

In addition to the 62 strains newly sequenced for this study, a large library of strains (approximately 220) previously published by our lab and others were downloaded from NCBI’s SRA. These strains were initially assessed for genome completeness after de novo assembly (see *De-Novo Assembly and Annotation methods* below), using BUSCO v4.0.5 [48] and coverage (read depth) using BBTools (https://jgi.doe.gov/data-and-tools/bbtools/) as well as phylogenetic diversity (visual inspection of redundant strains likely to be clonal—see *Phylogenomics* below for tree building methods). We then excluded strains with an average read depth <10×, or BUSCO complete scores <96, or with negligible branch lengths. Out of the approximately 220 strains initially downloaded, 197 strains were retained for analysis (S1 Table). These were combined with the 62 newly sequenced strains and the re-sequenced reference strain AF293, which was included as a control for variant analysis conducted relative to the Af293 v.55 curated reference downloaded from FungiDB. This resulted in a total of 260 quality-filtered strains.

### De-novo assembly and annotation

To minimize methodological errors introduced by analyzing genomes assembled and annotated using different methods [49], we ensured uniformity by assembling and annotating all 260 strains using a custom pipeline that starts with raw reads, regardless of whether assembly and annotation data were available for strains with previously released genomes. Genomes were assembled de novo with the Automatic Assembly For The Fungi (AAFTF) v. 0.2.3 (https://github.com/stajichlab/AAFTF, DOI: 10.5281/zenodo.1620526). The AAFTF *filter* step trim and quality filter reads with BBmap (https://sourceforge.net/projects/bbmap) and the AAFTF *assemble* step was used to assemble the reads with SPAdes [50]. Resulting contigs were screened for bacterial contamination using the AAFTF *sourpurge* step that relies on sourmash [51] searching a database of Genbank microbial sequence sketches (v.lca-mark2; https://osf.io/vk4fa/). Duplicates were removed with AAFTF *rmdup* by aligning contigs to themselves with minimap2 v. 2.17 [52], and contigs were further polished for accuracy using the AAFTF *polish* step that relies on Pilon v. 1.22 [53] and BWA v. 0.7.17 [54] to align raw reads to the contigs and polish a consensus sequence. Scaffolds from contigs were inferred by aligning to the reference Af293 genome using ragtag v. 1.0.0 [55]. Scaffolding to Af293 (currently the most complete reference genome for *A*. *fumigatus*) was carried out to improve scaffold length, but contigs failing to map to the reference were retained.

Repeat regions were masked by funannotate *mask* (https://github.com/nextgenusfs/funannotate, DOI: 10.5281/zenodo.1134477) using RepeatMasker v. 4-1-1 [56] with repeats built using RepeatModeler [57] compiled into a custom library (available in this the project’s GitHub) plus those fungal repeat families curated in RepBase v. 20170127 [58]. The *train* command in funannotate was used to run Trinity [59] for transcript assembly and alignments to generate highly polished gene models based on splice-site aware alignment of the assembled RNA-Seq of *A*. *fumigatus* growth on sugarcane bagasse (PRJNA376829) [60]. Models were further refined by PASA v. 2.3.3, and the best set with full open reading frames was chosen for input in training gene predictors [61]. Gene prediction was carried out using funannotate *predict* running Augustus v. 3.3.3 [62] and SNAP v. 2013-11-29 [63] by first training on the evidenced-based training models from the *train* step. Gene prediction was then run with these trained parameters using exon evidence based on RNA-seq and protein alignments generated by DIAMOND v. 2.0.2 [64] and polished with Exonerate v. 2.4.0 [65] on the RNA-seq and Swissprot proteins [66]. Additional ab initio models were predicted using GeneMark v. 4.59 [67], GlimmerHMM v. 3.0.4 [68], and CodingQuarry v. 2.0 [69], the latter also used the raw RNA-seq evidence for exon prediction directly. A set of consensus gene models were produced from these predictions with EVidenceModeler v. 1.1.1 [70]. The tool funannotate *annotate* was run to annotate protein domains and make functional predictions using sequence similarity with the databases InterProScan v. 5.45–80.0 [71], eggNOG v. 1.0.3 [72], dbCAN2 v. 9.0 [73], UniprotDB v. 2020_04, antiSMASH v. 5.1.2 [74], and MEROPS v. 12.0 [75].

### Pan-genomics

We used 2 methods to determine pan-genome gene family clusters: OrthoFinder v. 2.5.2 [76] and Pangenome Iterative Refinement and Threshold Evaluation (PIRATE) v. 1.0.4 [77]. The decision to assess the *A*. *fumigatus* pan-genome twice using 2 different methods was made to confirm the reproducibility of gene family counts after initial assessment yielded a surprisingly high number of orthogroups. OrthoFinder was run on protein fastA files with *-op* option to run similarity searches as parallel jobs on the HPC and the *-S diamond_ultra_sens* option for sequence search using DIAMOND. PIRATE was run on nucleotide fastA files, with parameters: *-s “85*,*86*,*87*,*88*,*89*,*90*,*91*,*92*,*93*,*94*,*95*,*96*,*97*,*98*,*99*,*100” -k “—cd-low 100 -e 1E-9—hsp-prop 0*.*5” -a -r*. Pan-genome clustering results were analyzed in the R programing environment using custom scripts (all scripts are available at the DOI listed in the data availability section). For our analysis, “core” genes were defined as present in >95% of the strains (*n* > 247), “accessory genes” were defined as present in more than 1, and less than 95% of the strains (*n* > 1 and < = 247) and singletons were defined as present in only a single isolate. Singletons and accessory gene families, collectively referred to here as “dispensable genes,” were analyzed separately to account for the possibility that singletons were more error prone or less likely to contain functional information. Significant differences in the abundance of accessory and singleton gene families per clade were assessed using a permutation test implemented in the R package *perm* [78] over 9,999 permutations to account for violation of variance assumptions. Gene family accumulation curves were calculated using the *specaccum()* function over 100 iterations in R package *vegan* v. 2.5.7 [79]. Analysis of clade-specific gene family absence was defined as a gene family absent in all isolates of that clade, but present in both of the other clades and in >90% of the isolates from at least one of those clades. Additional screening for clade-defining gene family absences was also conducted, defined as absent in all isolates of that clade, but present in >95% of all isolates from both of the other clades. Secreted proteins were predicted using the programs Signal P5 [80] and Phobius [81]. Phobius predictions were further refined by both the presence of a signal peptide and the absence of transmembrane domains. Significant differences in proportions of secreted gene families relative to all gene families were assessed using pairwise proportion tests with Bonferroni adjustment for multiple comparisons using function *pairwise*.*prop*.*test()* in the R *stats* package at *p* < 0.05.

### Functional analysis

Gene Ontology (GO) enrichment analysis was performed by assigning Interpro and GO annotations to the longest representative of each gene family clustered in OrthoFinder. GO enrichment analysis was then performed for each clade, on all gene families unique to that clade using the R package *topGO* v. 2.42.0 with the functions *new()* and *runTest()* with parameters *weight01* and *nodeSize = 6* (DOI: 10.18129/B9.bioc.topGO). Significant enrichment was assessed for each gene count category (core, accessory, singleton) and for clade-specific gene families relative to the set of all InterPro annotations with associated GO terms in the *A*. *fumigatus* pan-genome (*n* = 32,857), using a Fishers Exact test at *p* < 0.05. CAZyme (Carbohydrate-Active enZyme) annotations were assigned in funannotate that relies on hmmsearch to search the dbCAN database for HMM profiles [73]. Homogeneity of variance assumptions were checked using the *leveneTest()* function from the *car* package v. 3.0.11 and normalcy checked using the *shapiro*.*test()* function from the *stats* package v. 4.0.1 in R. Because variance and normalcy assumptions could not be met, nonparametric Kruskal–Wallis tests were employed using the *kruskal*.*test()* function in the R *stats* package v. 4.0.1 with Bonferroni adjustment for multiple comparisons using the *p*.*adjust()* function and considered for further evaluation at *p* < 0.001. Post-hoc comparisons using Dunn’s test were implemented using the *dunnTest()* function with Bonferroni adjustment for multiple comparisons in the R package *FSA* v. 0.9.0. CAZymes significantly different between the 3 clades were normalized per CAZyme family on a 0 to 1 scale for visualization and mapped onto the phylogeny using the *gheatmap()* function in the R package *ggtree* v.3.1.2. To validate our OrthoFinder clustered gene families, we represented each gene family with the longest sequence and used BLASTP v. 2.12.0 (e-val < 1e^-15^) to match gene families to their respective genes annotated in the Af293 reference genome. To further investigate the presence–absence distribution of accessory genes with a role in nitrogen, carbohydrate, and phosphorus metabolism, we extracted all Af293 genes from FungiDB with GO annotations that matched organonitrogen compound metabolic process (GO: 1901564, *n* = 1,775 genes), carbohydrate metabolic process (GO: 0005975, *n* = 467 genes), or organophosphate biosynthetic process (GO: 0090407, *n* = 215 genes). We then examined gene families with orthologues in Af293 to test for presence–absence variation in these genes between the 3 clades. Significant differences in gene abundance between the 3 clades were evaluated using the same strategy outlined above to evaluate CAZyme abundance.

### Spatial enrichment

To identify the location of core accessory and singleton gene families relative to telomeres, we calculated the enrichment of each gene family abundance category over 50 kb windows First,.gff files were converted to bed format and filtered to consider only contigs >50 kb in length and containing telomeric repeats (TAAC or the reverse complement) as developed by [82]. We then calculated the number of gene families contained either within or outside 50 kb of telomeric repeats using PyBedTools [83]. To test for enrichment of gene family abundance within 50 kb of telomeres, we checked normalcy assumptions using the *shapiro*.*test()* function in the *stats* package v. 4.0.1 in R, and as normalcy assumptions could not be met, used a 2-sided Wilcoxon rank sum test implemented in the *stats* package to test for significance.

### Population genomics

The Af293 reference genome was downloaded from FungiDB (v.46) [84]. Sequence reads for each strain were aligned to Af293 using BWA v. 0.7.17 and the alignment file processed with samtools v. 1.10 [85], applying the *fixmate* and *sort* commands to convert files to the BAM format. Duplicate reads were removed, and reads were flagged using *MarkDuplicates* and indexed with *Build BamIndex* in picard tools v.2.18.3 (http://broadinstitute.github.io/picard). Variants (SNPs) were called relative to Af293 using *HaplotypeCaller* in GATK v.4.0 [86] with filtering accomplished using GATK’s *VariantFiltration*, with parameters *-window-size = 10*, *-QualByDept <2*.*0*, *-MapQual <40*.*0*, *-Qscore <100*, *-MapQualityRankSum <-12*.*5*, *-StrandOddsRatio > 3*.*0*, *-FisherStrandBias >60*.*0*, *-ReadPosRankSum <-8*.*0*. Only variants passing these filers were retained using the SelectVariants tool in GATK. Variants overlapping TEs were further excluded from the variant pool by testing for overlap with the TE locations identified in the FungiDB v. 46 release of the Af293 reference genome, using bedtools *subtract* [87]. Variants were annotated with snpEff [88] based on the GFF annotation for Af293 v. 46 from FungiDB.

### Population structure

Prior to analysis, the presence of clones in the dataset was assessed using the R package poppr v.2.9.0 [89] with the functions mlg() and clonecorrect(). No clones were detected. Initially, we assessed broad-scale population structure in *A*. *fumigatus* using the Bayesian clustering approach STRUCTURE implemented in fastStructure v. 1.0 [90] across 361,717 polymorphic sites (single nucleotide polymorphisms). VCF files were first converted into the plink format using PLINK v. 1.90b3.38 [91] before running fastSTRUCTURE using the simple prior with K values ranging from 1 to 15 over 30 independent iterations with specified seed values. For each independent iteration, marginal likelihood values of K were obtained by employing the *chooseK*.*py* function in fastSTRUCTURE and assessed for significant increases in marginal likelihood using ANOVA and the R package *multcomp* [92] with post hoc testing using Tukey tests and Bonferroni correction for multiple comparisons. To further assess population structure, we used discriminate analysis of principle components (DAPC) [93] and subsequent clade mapping onto the phylogeny. DAPC was implemented in the R package *adegnet* v. 2.1.3 [94] on a random subset of 100 K polymorphic SNP sites. The optimal number of groups (K) was identified based on Bayesian information criterion (BIC) score using the *find*.*clusters()* function in *adegnet*, evaluating a possible range of 1 to 15 clusters to identify the elbow of the BIC curve following [93]. To avoid overfitting, the optimal number of PCs retained in the DAPC was chosen using the *optim*.*a*.*score()* function and determined to be PC = 3 out of a possible PC range of 1 to 200. Evaluation of population substructure was carried out by iteratively running DAPC and fastSTRUCTURE on the 3 primary clades identified above. To do this, clade-specific VCF files were subset using VCFtools—*keep* [95], and invariant sites were removed using the bcftools *-view* command [96], before running the pipeline as above. To further investigate the history of gene flow in *A*. *fumigatus*, we ran TreeMix [97]. TreeMix was run on a VCF file containing all strains plus *A*. *fischeri* as the outgroup on a VCF file prepared as above except with the addition of *A*. *fischeri*. Initially, the assembly for *A*. *fischeri* strain NRRL 181 genome was downloaded from FungiDB v. 55. The assembly was then used to simulate Illumina paired-end reads with the *wgsim* tool in the samtools package with an error rate of 0 and to a read depth approximately equal to the average for the *A*. *fumigatus* strains, and run as above. To run TreeMix, we removed invariant sites from the VCF as above, as well as sites with high LD using Plink v.2 with the parameter—*indep-pairwise 50 10 0*.*2*. To determine the optimal number of migration edges (m), we used the program OptM [98]. First, we prevented bootstrapped TreeMix runs from having an SD = 0 by down sampling the LD-corrected VCF file to 80% of total SNP sites, randomly drawn over 10 replicates using the bcftools *-view* command. Subset VCF files were converted to TreeMix format and for each down sampled replicate TreeMix was run for all values of m between 1 and 10, and used as input for OptM in R. We then re-ran TreeMix at the optimal value of m = 1 using the full LD-corrected VCF file as input, with all trees rooted at *A*. *fisheri*, using *A*. *fisheri* as the outgroup and the *A*. *fumigatus* subpopulations defined by iterative DAPC analysis as described above. The fixation index (F_ST_) was calculated in the R package hierfstat v.0.5.9 [99] using the dosage format to account for unequal population sizes [100] and computed with the *fst*.*dosage()* function. Pairwise F_ST_ (the between population fixation index) was calculated in hierfstat using the *fs*.*dosage() $Fst2x2* function. As a second measure of population differentiation, we ran analysis of molecular variance (AMOVA) using the R packages *poppr* and *ade4* [101] with the function *poppr*.*amova()* with *method = “ade4*.*”* Significant differentiation between populations was evaluated using a Monte Carlo test with the function *randtest()* in *poppr*, over 999 iterations. To distinguish introgression from incomplete lineage sorting, we calculated Patterson’s D (ABBA-BABA) in the program Dsuite using the Dtrios tool [102]. Dtrios was run on all 260 strains, set to 3 populations, with population membership encodings determined in the previously reported DAPC analysis, plus *A*. *fischeri* as the outgroup. Significance of the deviation of the D-statistic from zero was determined using a 20 block-jackknife approach with a *p*-value <0.05 indicating introgression.

### Linkage disequilibrium

To estimate linkage disequilibrium (LD) decay, the VCF SNP files (run without *A*. *fischeri*) generated above were used in conjunction with the clade assignments generated in DAPCA for K = 3 to assess LD decay using PLINK [91]. LD decay was assessed both for all isolates together and for all isolates separated by clade. Additionally, we assessed LD decay on a sample size rarified set of isolates at *n* = 12 averaged over 20 independent runs. To do this, the samples were rarefied using a custom BASH script, where for each clade, random draws of 12 isolates per clade were taken over 20 iterations. Sampling did not allow for the same strain to appear more than once in a single iteration, but did allow sampling with replacement between iterations. To specifically evaluate the impact of sample size on LD estimates, we also ran the analysis for *n* = 5, 10, 20, 50, or 100 isolates (averaged over 20 independent runs) randomly drawn without regard to population structure. For all analyses, strains were subset (when appropriate) from the VCF file as noted above, using vcftools—*keep* and bcftools—*view* before converting VCF files to PLINK format using PLINK v. 1.90b3.38. LD decay was estimated in PLINK using the squared genotypic correlation coefficient (r^2^) over all SNPs present in each group. We considered up to 99,999 variants per window and all r^2^ values to ensure fine scale resolution. Pairwise distance comparisons were limited to 500 kb (with parameters:—r2 –allow-extra-chr–ld-window-r2 0 –ld-window 99999 –ld-window-kb 500). Distance matrices were constructed in BASH and mean r^2^ for each distance was assessed across each iteration, and then across each group to generate LD decay curves for each group using *ggplot2* [103]. To estimate half-decay values (LD50) in base pairs (BP) for each dataset, we averaged the r^2^ for each position across all replicates in each group and calculated the r^2^ mid-point as (minimum r^2^ + (maximum r^2^ –minimum r^2^) / 2). To obtain LD50 in BP, we then calculated the x intercept of the r^2^ mid-point for each clade using the *approx()* function in R.

### Phylogenomics

The identified alleles for SNP positions from all isolates were used to construct a phylogeny employing the Maximum Likelihood algorithm IQ-TREE using the *+ASC* parameter to account for SNP-based ascertainment bias [104]. The best fit model according to BIC score was assessed using the *ModelFinder* function in IQ-TREE and was determined to be GTR+F+ASC that was run over 1,000 rapid bootstrap iterations. Individual branch support values were assessed using a Shimodaira–Hasegawa approximate likelihood ratio test over 1,000 iterations. Tree rooting was determined by evaluation of the SNP tree run as above but including the outgroup *A*. *fischeri*. Based on outgroup position, the root was determined to be at the basal node separating Clades 2 and 3. Tree visualization and trait mapping was carried out using the R package *ggtree* [105].

### Distribution of mating type idiomorphs

To identify MAT type for each strain, we downloaded reference CDS DNA sequences for MAT1-1 (GenBank number AY898661.1 from strain AF250) and MAT1-2 (Afu3g06170, NCBI number NC_007196.1 from strain Af293) from NCBI. We constructed databases composed of all scaffolds for each genome, and ran BLASTN [106] with the parameters *-evalue*. *0001 and -word_size 10*, against both MAT idiomorphs. BLAST hits were formatted as fastA files using a custom BASH script and aligned using MAFFT v. 7.471 [107]. To ensure that strains with significant alignments to both MAT1-1 and MAT1-2 were not artifacts in the de novo assembled scaffolds, these strains (*n* = 11) were further evaluated by aligning the raw reads onto the MAT reference sequences. To accomplish this, reference sequences were indexed using Bowtie2 v. 2.3.4.1 [108] with the bowtie2-build function, and SAM files created from alignments generated with the *-very-sensitive-local* option. SAM files were converted to BAM using the samtools *view* function and sorted using the *sort* function. Depth profiles of the alignments were created using the samtools *depth* function and graphed in R using the package ggplot2. To create fastA files of the alignments, we used the bcftools *mpileup* function to create VCF files, indexed the VCF files using bcftools *tabix*, and used samtools *faidx* to index the reference sequences. Then, using picard v. 2.18.3, we used the *CreateSequenceDictionary* function to insert variant information for each strain into the reference MAT sequences and the bcftools *consensus* function to insert “N”s for all positions where no alignments could be made. To assess the ploidy of these 9 strains, we used whole-genome K-mer analysis on forward reads implemented in the program Jellyfish [109], and visualized using GenomeScope [110], at K = 21. To further assess the ploidy of these 9 strains, we conducted allele frequency analysis on all heterozygous SNP sites aided by the R package *vcfR* [111]. Identification and mapping of the MAT1-2-4 gene (Afu3g06160) was conducted using BLASTP matches to Af293 gene assignments to the orthogroup clusters and mapped onto the phylogeny as above.

### Presence–absence variance and SNP distribution of antifungal resistance genes and virulence factors

A database of characterized *A*. *fumigatus* antifungal resistance variants was assembled from the literature and combined with all MARDy database entries for *A*. *fumigatus* (http://mardy.dide.ic.ac.uk) (S2 Table). We analyzed the population frequency of characterized resistance-related amino acid changes available for 6 genes, as well as surveying all amino acid changing variants across these 6 genes and an additional 7 genes associated with azole resistance in the literature, but for which no regulatory amino acid changes have been characterized. Population frequency of amino acid changing variants was conducted using custom scripts in R and mapped onto the phylogeny using the packages *ggplot2* and *ggtree*. We used the BLASTP matches to Af293 gene assignments to validate the credibility of clade-specific gene family assignments and to look for presence–absence variation in *A*. *fumigatus* secondary metabolite biosynthetic gene clusters, consisting of 230 genes across 26 clusters as defined in [112]. To determine the boundary of a large deletion in the gliotoxin cluster covering *gliI*, *gliJ*, and *gliZ*, in some strains, we extracted the genes up and downstream from the de novo assemblies, Afu6g09690 and Afu6g09660 (*gliP*), using genome coordinates as defined for the Af293 reference genome. These sequences were then aligned onto Af293 using minimap [113]. To visualize the boundary of the deletion, a truncated example of *gliP* was extracted using samtools *faidx* (from AF100-12_9) using the coordinates identified in the alignment, and DNA was translated for both reference and truncated versions of the *gliP* gene using exonerate [65]. Architecture was modeled using domain annotations for *gliP* from the Af293 reference in UniProt [114] and visualized using the R package *drawProteins* [115].

## Results

### Genome sequencing and assembly statistics

Average sequencing depth per strain ranged from 12X coverage (IFM_59356–2) to 359X coverage (MO91298SB) with a mean depth of 75X. BUSCO scores ranged from 96.3% (MO91298SB) to 99.4% (16 strains) completeness with a mean of 98.9%. The total number of scaffolds ranged from 38 (Afum_84-NIH) to 872 (AF100-1_3) with a mean of 191. Scaffold L50 ranged from 3 to 4, with a mean of 3.9. Scaffold N50 ranged from 3.53 Mbp (megabase pairs) to 4.35 Mbp with a mean of 3.94 Mbp. Genome size ranged from 27.438 Mbp (CM2495) to 31.568 Mbp (NCPF-7820) with a mean of 29.128 Mbp (S1 Table).

### Population structure

**Fig 1:**
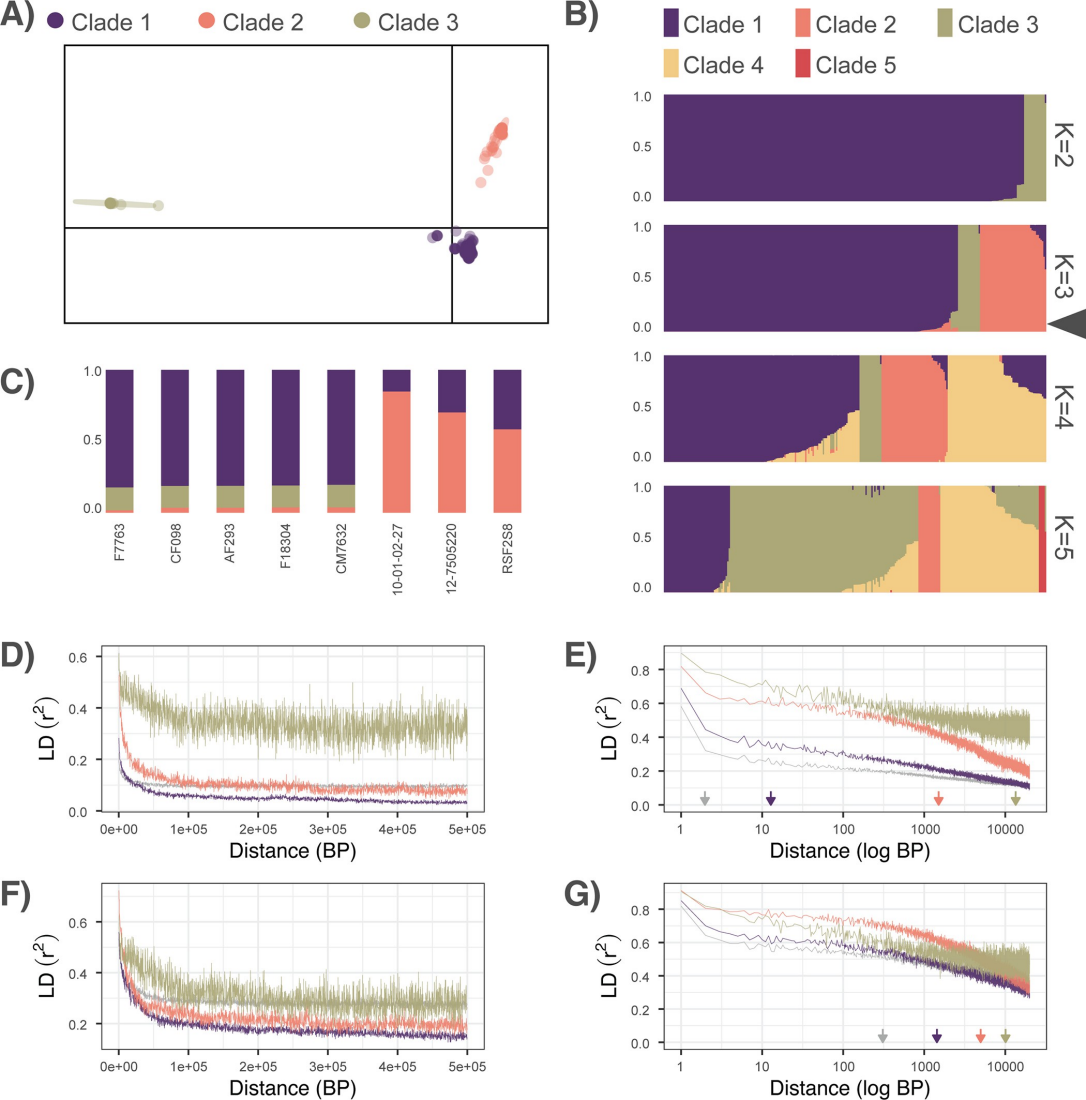
** (A)** DAPCA plot depicting 3 primary populations with clear separation between clusters. The x-axis represents the first discriminant function, y axis represents the second discriminant function, ellipses represent 95% confidence areas. **(B)** STRUCTURE plots of K = 2 to K = 5 populations support low levels of admixture between clades at K = 3 (highlighted with triangle). **(C)** Isolates with <0.85 probability of assignment to a single clade, indicative of admixture or incomplete lineage sorting between clades. **(D, E)** LD decay (r^2^) for all isolates in each respective clade or all isolates across all clades (in gray). **(D)** Linear–linear plot of LD decay. **(E)** Zoomed-in log-linear plot of LD decay, with arrows indicating LD50 (half decay) values. **(F, G)** LD decay (r^2^) for sample size normalized isolates (*n* = 12 isolates, averaged over 20 draws) in each respective clade or for all isolates across all clades (in gray). **(F)** Linear–linear plot of LD decay. **(G)** Zoomed-in log-linear plot of LD decay, with arrows indicating LD50 values. The data underlying this figure can be found in DOI: 10.5281/zenodo.5775265. BP, base pair; LD, linkage disequilibrium.

To assess population structure and the frequency of recombination overall, within, and between the clades, we used a combination of fastSTRUCTURE, DAPC, population statistics, and LD decay analysis. fastSTRUCTURE’s marginal likelihood values increased until K = 5, but did not increase significantly after K = 4 with a mean marginal likelihood difference of only 0.0005 between K = 3 and K = 4 (S1 Fig). Because fastSTRUCTURE tends to overestimate K when K is small [90] and is predicated on the assumption of locus independence due to recombination, which is violated by clonality, we further defined K using DAPC analysis. The optimal K was approximately 3 (S2A Fig), using the BIC criteria with 3 principal components retained (S2B Fig). DAPC supported clear separation of 3 primary clades (Fig 1A). Clade 1 was the largest with 200 strains, followed by Clade 2 with 45 strains, and Clade 3 with 15 strains (S3 Table). According to DAPC, none of the strains in this study met the criteria for admixture (membership coefficients <0.85), as membership coefficients were essentially 1 in all cases (S3 Table). While clade assignment to 1 of the 3 clades using fastSTRUCTURE placed all strains in the same respective clades as DAPC, fastSTRUCTURE membership coefficients were lower overall than DAPC membership coefficients (Fig 1B and S4 Table) and a total of 8 strains had membership coefficients <0.85, indicative of admixture or incomplete lineage sorting between 2 or more of the clades (Fig 1C). These included 3 strains (RSF2S8, 12–7505220, and 10-01-02-27) assigned to Clade 2 but with ancestry from Clade 1 and 5 strains (F7763, CF098, F18304, CM7632, and the resequenced reference strain AF293) assigned to Clade 1, but with ancestry from both Clades 2 and 3. To investigate population substructure within the 3 clades, we used iterative DAPC and fastSTRUCTURE analysis on each clade separately. Subset VCF files excluding all invariant sites consisted of a total of 61,618 SNPs in Clade 1, 132,130 SNPs in Clade 2, and 155,585 SNPs in Clade 3. The difference in SNP abundance between the 3 clades is consistent with the location of the reference Af293 strain in Clade 1. The optimal number of PCs for the sub-clades was determined to be 5 for the substructure within Clade 1 (S3A Fig) and 3 for the substructure within Clade 2 (S3B Fig). The optimal number of PCs for Clade 3 was determined to be 1 (S3C Fig) and was therefore not further analyzed with DAPC. Iterative DAPC analysis supported 5 sub-clades within Clade 1 and 5 sub-clades within Clade 2 (S3D and S3E Fig and S5 Table). DAPC and fastSTRUCTURE returned slightly different clade memberships for sub-clades at K = 5 (S5 and S6 Tables). fastSTRUCTURE analysis at K = 5 supported frequent introgression between sub-clades in both Clade 1 (87 out of 200 strains) (S3F Fig) and Clade 2 (7 out of 45 strains) (S3G Fig).

Mapping the 3 clades onto the phylogeny was congruent with clade membership, with all clades demonstrating monophyly, with the exception of all of the Clade 1 strains with signatures of admixture, which clustered on their own branch between Clades 2 and 3. Similarly, the optimum value of migration edges according to OptM was determined to be 1 (S4A and S4B Fig), with TreeMix placing the migration vector from Clade 3 to sub-clade Clade 1.1, containing the Clade 1 isolates with signatures of introgression (S4C Fig). Overall, F_ST_ was 0.824, and within population F_ST_ was 0.767, 0.825, approximately 0.880 for populations 1, 2, and 3, respectively. Pairwise (between population), F_ST_ was 0.531 between Clade 1 and 2, 0.887 between Clade 2 and 3, and 0.859 between Clade 1 and 3. Similarly, AMOVA supported that the majority of variation, 69.8%, occurred between populations (σ = 45,009.85, *p* = 0.001), whereas differences between individuals in the populations accounted for only 30.2% of the total variation (σ = 19,482.91). The value of Patterson’s D was 0.135 (Z = 24.32, *p* < 0.0001, Dmin = 0.118), indicating introgression over incomplete lineage sorting. LD50 estimates were extremely low when evaluated without considering clade or sample size (1.97 BP considering all 260 isolates) and notably higher when taking clade into account (12.79 BP for Clade 1, 1,508.32 BP for Clade 2, and 13,474.24 BP for Clade 3, Fig 1D and 1E). Modeling the influence of sample size on LD estimates showed a strong inverse relationship between sample size and LD (S5 Fig). Similarly, sample size normalized LD estimates at *n* = 12 were notably higher than without normalization, consistent for the overall LD (LD50 = 308.00 BP) and for the LD of Clades 1 (LD50 = 1,425.24 BP) and 2 (LD50 = 4,974.58 BP), but not for the smallest and most closely related clade, Clade 3 (LD50 = 10,096.73 BP) (Fig 1F and 1G).

### Pan-genome

**Fig 2:**
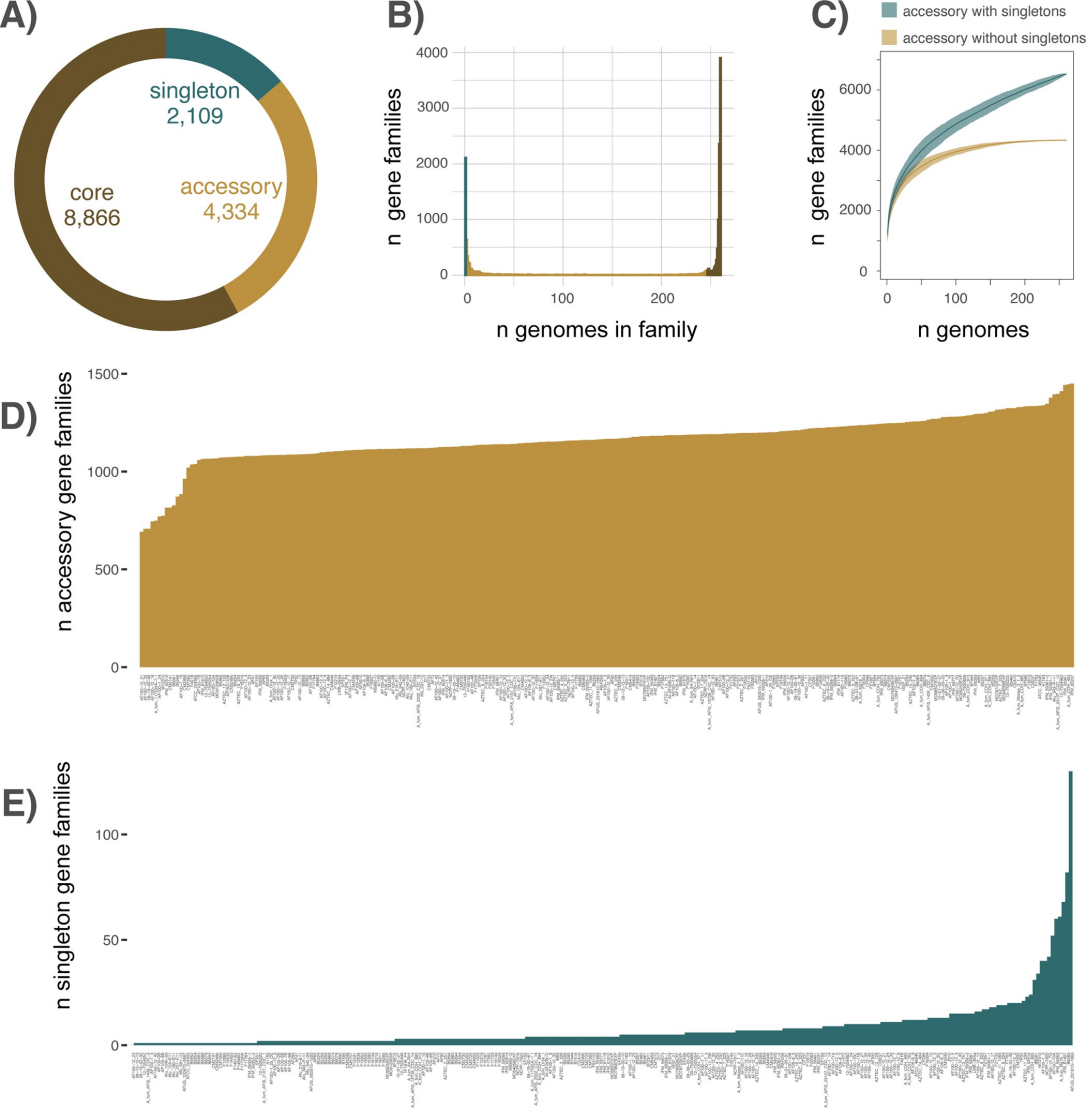
Pan-genome gene family distribution. **(A)** The pan-genome of 260 *A*. *fumigatus* strains included 15,508 gene families in total, including 8,595 (55.42%) core genes (present in >95% of strains), 3,660 (23.60%) accessory genes (present in >2 and <248 strains), and 3,253 (20.98%) singletons (present in only 1 isolate). **(B)** The distribution of the number of genomes represented in each gene family. **(C)** Gene family accumulation curves, including (green) and excluding (yellow) singletons. **(D)** The distribution of unique accessory gene families by strain. **(E)** The distribution of unique singleton gene families by strain. The data underlying this figure can be found in DOI: 10.5281/zenodo.5775265.

**Fig 3:**
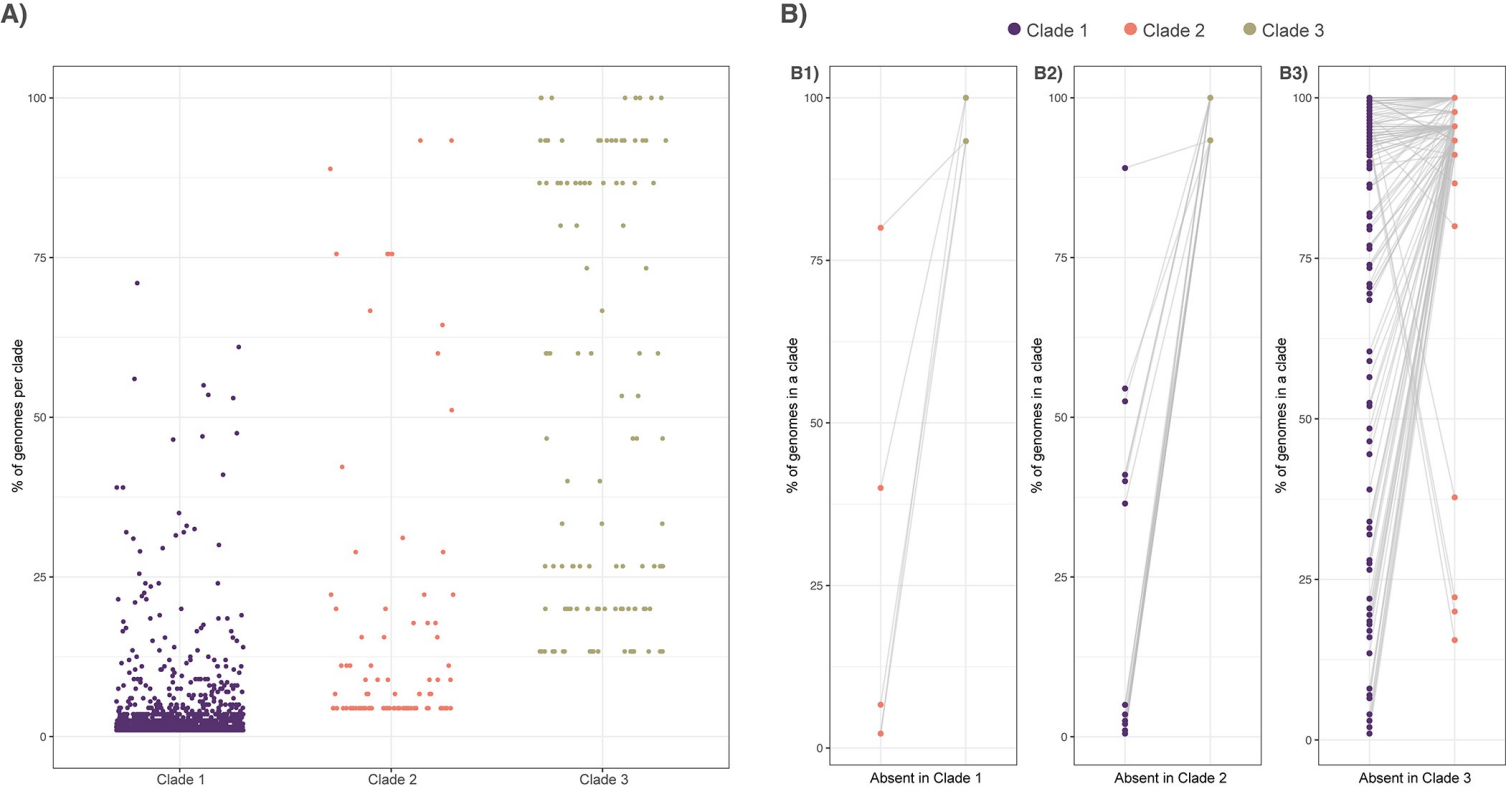
Distribution of clade-specific gene family gains and absences. **(A)** The occurrence of clade-specific gene families in that clade (x-axis), defined as genes that exist in that clade in more than 1 genome (singletons were excluded), by prevalence in that clade (y-axes), and highlights that while many clade-specific gene families that exist in a low number of isolates, others are present in nearly all the isolates of that clade and in no other isolates. The distribution of occurrence frequency varies between the 3 clades, due to differences in clade size, with 1,256 accessory gene families exclusive to Clade 1, 95 exclusive to Clade 2, and 115 exclusive to Clade 3. **(B)** The occurrence of clade-specific gene family absences for clade-defining absences, where each panel depicts the occurrence of each gene in the 2 clades where the gene is not absent. The distribution of each gene is connected by a gray line. **(B1)** Gene families lost in all isolates of Clade 1 but present in greater than 90% of the isolates in either Clade 2 or Clade 3 (*n* = 5). **(B2)** Gene families lost in all isolates of Clade 2 but present in greater than 90% of the isolates in either Clade 1 or Clade 3 (*n* = 25). **(B3)** Gene families lost all isolates of Clade 3 but present in greater than 90% of the isolates in either Clade 1 or Clade 2 (*n* = 125). The data underlying this figure can be found in DOI: 10.5281/zenodo.5775265.

In total, we identified 15,309 gene families including 8,866 core genes (57.91% of the total), 4,334 accessory genes (28.31%), and 2,109 singletons (13.78%) using OrthoFinder (Fig 2A), and a total of 15,476 gene families, including 8,600 core genes (55.57% of the total), 3,618 accessory genes (13.92%), and 3,258 singletons (21.05%) using PIRATE (S6A Fig). The distribution of the number of genomes represented in each gene family was characteristically U-shaped and ranged between 1 and 260 for both OrthoFinder (Fig 2B) and PIRATE identified gene families (S6B Fig). Gene accumulation curves yielded conflicting genome structures where accessory gene families excluding singletons leveled after sampling approximately 150 genomes (suggesting a closed pan-genome) but did not reach saturation (suggesting an open pan-genome) when considering singletons along with accessory genes for both OrthoFinder (Fig 2C) and PIRATE identified gene families (S6C Fig). The number of unique accessory gene families per strain from OrthoFinder identified gene families ranged from 692 in AF100-12_21 to 1,450 in IFM_60237 with an average of 1,163 per strain (Fig 2D). PIRATE identified gene families ranged from 665 in AF100-12_21 to 1,376 in IFM_60237 with an average of 974 per strain (S6D Fig). The number of unique singleton gene families per strain ranged from 1 (34 strains) to 130 in the strain AFUG_031815_1869, with an average of 8 per strain. One strain (AF100_12_23) contained no singleton gene families (Fig 2E). PIRATE identified gene families supported singleton gene families ranging from 1 (27 strains) to 191 (in AFUG_031815_1869) with an average of 13 per strain and 45 strains with no singleton gene families (S6E Fig). Although AFUG_031815_1869 was an outlier in the number of singletons present in both the OrthoFinder and PIRATE analysis, all AFUG_031815_1869 genome quality metrics were average relative to the rest of the dataset (200 scaffolds, average depth of 53 BP, and a BUSCO score of 99.3), and it did not appear that accessory genes were being miscategorized as singletons, so we elected to include this strain in all downstream analysis. Overall, both accessory (*p* = 8.3e^-8^, R^2^ = 0.12) and singleton *p* = 4.9e^-5^, R^2^ = 0.06) gene families were significantly related to genome size, although with low R^2^ values, particularly for singleton gene families (S7 Fig). Neither the number of accessory gene families per strain nor the number of singleton gene families per strain was significantly different between clades, with means of 1,171, 1,160, and 1,075 accessory gene families per genome in Clades 1, 2, and 3, respectively, and 9, 4, and 8 singleton gene families per genome for OrthoFinder identified gene families (*p* = 0.42 for accessory gene families and *p* = 0.49 for singleton gene families). These results were largely consistent for PIRATE identified gene families, with means of 980, 973, and 909 accessory gene families per genome in Clades 1, 2, and 3, respectively, and 14, 6, and 16 singleton gene families per genome, and no significant differences in gene family abundance between clades. Because of the relatively similar results obtained between OrthoFinder and PIRATE, we conducted the remainder of the analysis solely on the OrthoFinder identified gene families (hereafter referred to as just gene families). Clade 1 contained 1,256 unique accessory gene families (not found in the other 2 clades) (average 6 per strain), Clade 2 contained 95 unique accessory gene families (average 2 per strain), and Clade 3 contained 115 unique accessory gene families (average 8 per strain) (Fig 3A). Clade-defining gene family gains (defined as present in >90% of strains in the clade, but in 0 strains from the other 2 clades) were 0 for Clade 1, 2 for Clade 2, and 23 for Clade 3. To account for the potential of introgressed strains to influence the binning of core, accessory, and singleton gene families and the identification of clade-specific gene families, we removed the 8 strains with evidence of introgression between the 3 primary clades and re-calculated gene family abundance. We found that excluding introgressed strains only slightly changed the overall gene family abundance categories: core gene families = 8,890, an increase of 24; accessory gene families = 4,246, a decrease of 88; and singleton gene families = 2,081, a decrease of 28. Similarly, excluding introgressed strains resulted in a slight decrease in the number of clade-specific gene families in Clade 1 (*n* = 1,248, a decrease of 8 gene families) and a slight increase in Clades 2 and 3 (*n* = 97 in Clade 2, an increase of 2 and *n* = 125 in Clade 3, an increase of 10). Analysis of clade-specific gene family absences identified 24 gene families missing in Clade 1 (with 5 clade-defining absences, defined as missing in all isolates of Clade 1 but present >90% of all isolates from Clades 2 or 3) (Fig 3B), as well as 270 gene families absent in Clade 2 (with 25 clade-defining absences), and 991 gene families absent in Clade 3 (with 125 clade-defining absences).

The proportion of secreted proteins was significantly higher in the core gene families than for accessory or singleton gene families (pairwise proportion test at *p* < 0.05). No significant differences were observed in the proportion of secreted proteins between the 3 clades (S8 Fig). Significant BLASTP hits for representative gene family sequences were found for 9,512 of the 9,840 genes currently annotated in the Af293 reference genome. While 9,299 gene families were represented by a single BLAST hit to the Af293 reference genome, the remaining 213 gene families had more than 1 significant hit, suggesting that some closely related gene families may have been merged into a single orthogroup. To investigate the potential for the over-splitting of gene families, we conducted a clade-wise test of diversity: Because the Af293 reference strain is a member of Clade 1, clade-specific gene families in Clades 2 and 3 should not be present in our BLAST searches if they are truly clade specific. Clade-specific gene families present in Clade 2 had no significant BLAST hits to the reference strain, but Clade 3 specific gene families had 2 gene families with significant matches to genes in the Af293 reference (Afu8g01650 and Afu3g02860—both encoding proteins of unknown function), indicating low levels of sequence similarity among genes that our approach classified as unique families. Clade-specific gene families in Clade 1 had 28 gene families that could be assigned an Af293 reference gene ID, all other Af293 annotated gene families were present in more than 1 clade (S7 Table).

### Distribution of mating type idiomorphs

**Fig 4:**
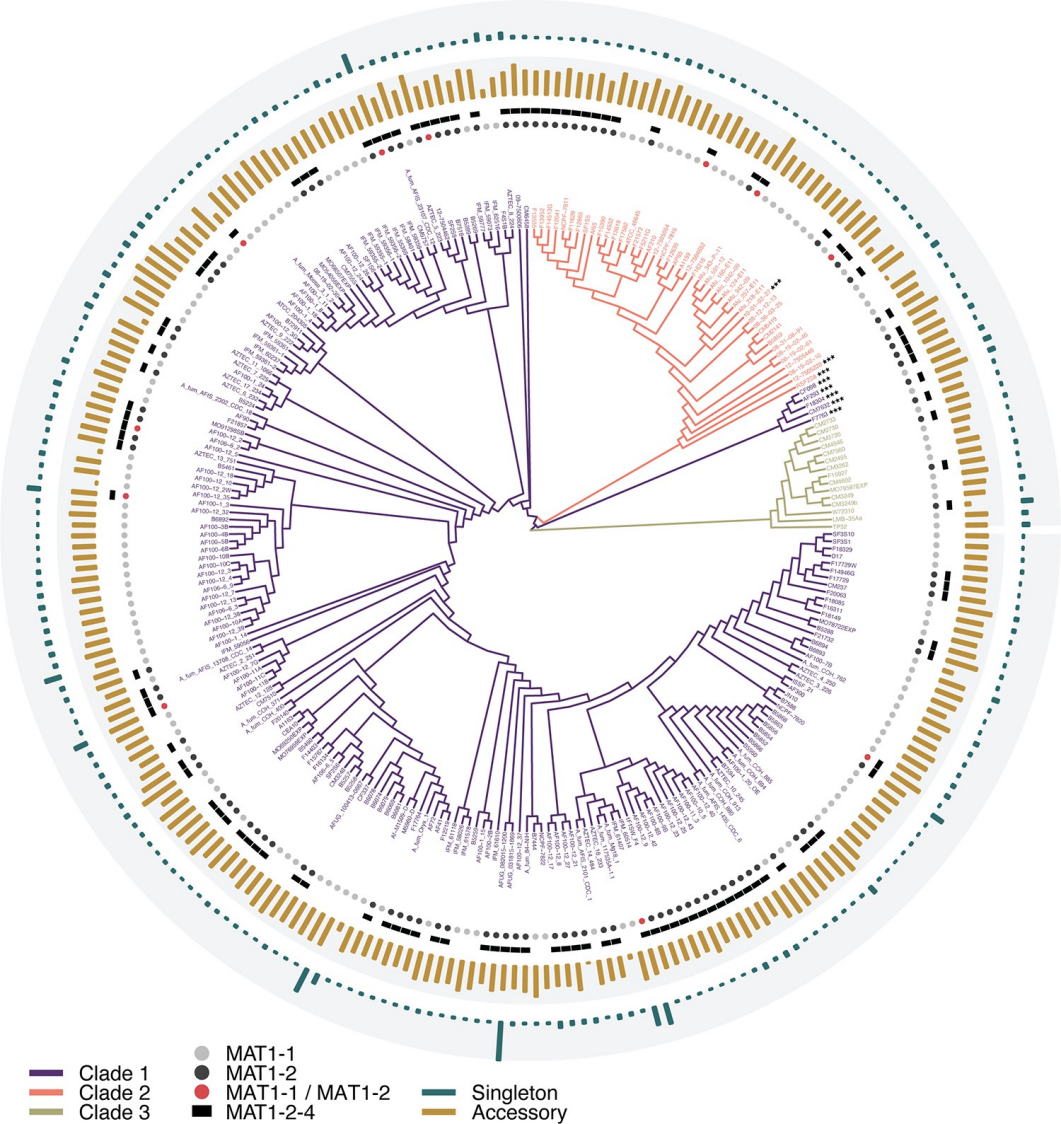
Phylogeny of *A*. *fumigatus* mapped against pan-genome distribution and MAT type. Overall abundance of accessory and singleton gene families was not structured by clade. The distribution of MAT type was random across the phylogeny, with both mating types present in all 3 clades. MAT idiomorphs were not present in equal proportion, with 144 strains containing the MAT1-1 idiomorph, 105 containing MAT1-2, and 11 strains presenting significant alignments to both MAT1-1 and MAT1-2. All strains with the MAT1-2 idiomorph also contained the MAT1-2-4 gene, including the 11 strains with alignments to both MAT1-1 and MAT 1–2. Strain names with the *** suffix denote the 8 strains demonstrating significant admixture between 2 or more clades. The data underlying this figure can be found in DOI: 10.5281/zenodo.5775265.

To determine the capacity for sexual recombination across the *A*. *fumigatus* phylogeny, we characterized the mating type of each strain relative to population structure. Whereas the MAT1-1 idiomorph has a unique sequence structure encoding the α-box domain, MAT1-2 contains both a unique region encoding a high-mobility group (HMG) region and a region conserved between mating types [116]. Additionally, MAT1-2 strains are expected to contain the gene MAT1-2-4 (Afu3g06160), essential for sexual recombination [117]. We therefore expected MAT1-1 strains to have full-length alignments to the MAT1-1 reference and a truncated alignment to the MAT1-2 reference, and MAT1-2 strains to have full-length alignments to the MAT1-2 reference, no alignment to the MAT1-1 reference, and the presence of MAT1-2-4. Using these criteria, we identified 144 isolates in our set with the MAT1-1 idiomorph and 105 isolates with the MAT1-2 idiomorph (Fig 4), a ratio of 48:35. Additionally, 11 isolates had significant alignments to both MAT1-1 and MAT1-2, hereafter referred to as “unknown” mating type. All unknown mating types also contained the MAT1-2-4 gene. All 3 clades contained both MAT types, in MAT1-1:MAT1-2 ratios of 107:85 (8 unknown) in Clade 1, 26:16 (3 unknown) in Clade 2, and 11:4 (0 unknown) in Clade 3. The 11 isolates with unknown mating type were further examined using read mapping and read depth analysis, which confirmed that for each of the 9 strains, raw reads did indeed map onto the full length of both reference idiomorphs, however, alignment depths of MAT1-1 relative to MAT1-2 differed among strains, and only 3 strains displayed relatively equal depth profiles for both MAT-1 and MAT-2 (AF100-1_3, IFM_59359, and IFM_61407) (S9 Fig). To investigate the possibility that these 11 strains represented diploids, we first conducted whole-genome K-mer analysis and found that 9 of the 11 strains had strong single peaks supporting haploidy, while 2 strains, AF100-1_3 and IFM_59359, had moderate secondary peaks, potentially indicative of diploidy (S10 Fig). To further assess strain ploidy, we conducted allele frequency analysis on all heterozygous SNPs and found that 3 strains (AF100-1_3, IFM_59359, and IFM_61407) displayed notable peaks at 1/2 frequency, lending further support to the potential for diploidy (S11 Fig). Three strains (08_36_03_25, NCPF_7816, and SF2S9) had no significant peaks at 1/2 allele frequency, but did display moderate shoulders sloping down from 1 (representing SNPs with base calls different than the Af293 reference) and up to 0 (representing SNPs with the same base call as the reference) potentially indicative of noise in the sequencing (miscalls, contamination, or tag switching), low levels of copy number variation, or segmental duplication. The remaining 5 strains (AF100-1_18, AF100_12_5, Afu_343_P_11, B7586_CDC_30, and AF100_12_7G) displayed only low levels of 1/2 allele frequency but did display paired peaks below 1/4 and above 3/4, indicative of haploidy with large-scale copy number variation or segmental duplication in these strains [118].

### Functional analysis

**Fig 5:**
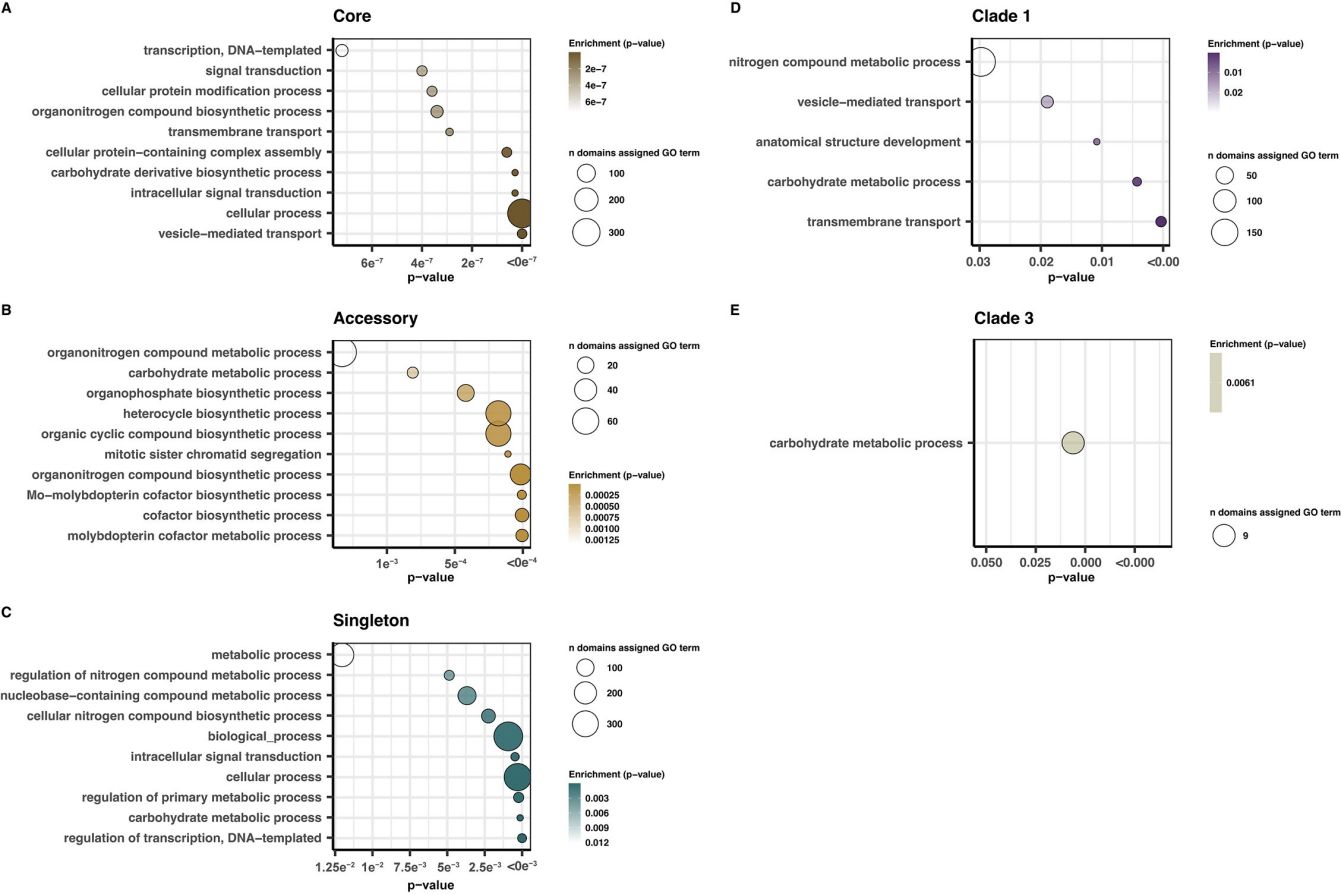
GO enrichment. Significantly enriched Biosynthetic Process GO terms associated with accessory gene families present exclusively in different *A*. *fumigatus* pan-genome categories **(A–C)** and clades **(D, E)**. Enrichment analysis was conducted for each experimental set against a background of all GO terms for all gene families in the *A*. *fumigatus* pan-genome; *p*-values represent results of Fisher’s exact test of the top 10 most significantly enriched terms (for terms enriched at *p* < 0.05) **(A)** core, **(B)** accessory, **(C)** singleton, **(D)** Clade 1, **(E)** Clade 3. Clade 2 had no significantly enriched terms. The data underlying this figure can be found in DOI: 10.5281/zenodo.5775265. GO, Gene Ontology.

To investigate the functional implications of pan-genomic variation across the 3 clades, we performed enrichment analysis both de novo and relative to the Af293 reference genome. While only 1.75% of core gene families were unable to be assigned with any functional annotation, this number was greater for accessory (20.52%) and singleton (18.73%) gene families. The percent of gene families unable to be assigned functional annotation was similar between Clades 1 and 2 (18.47% and 18.95%, respectively) but higher for Clade 3 (39.13%). GO enrichment analysis of gene families found exclusively in the core, accessory, and singleton categories identified significant GO terms in all 3 categories. The same analysis targeting clade-specific gene accessory gene families, identified terms significantly enriched in Clades 1 and 3 (Fig 5). Clade 2 was not significantly enriched for any GO terms. Core gene families were generally enriched for terms related to housekeeping functions like transport, signal transduction, and general cellular processes (Fig 5A). Accessory gene families were enriched in terms for nitrogen, carbohydrate, and phosphorus metabolic processes, with a small number of terms associated with molybdoprotein metabolic processes (Fig 5B). Singleton gene families were enriched for terms relating to carbohydrate and nitrogen metabolism, as well as primary metabolism and transcriptional regulation (Fig 5C). The most significantly enriched terms in Clade 1 also included terms associated with metabolism, including carbohydrate and nitrogen processing, as well as gene families associated with transmembrane transport and vesicle mediated transport (Fig 5D). There was only 1 term significantly enriched in Clade 3, also for carbohydrate metabolism (Fig 5E).

To further investigate metabolic genes differentially abundant between the 3 clades, we used gene families with Af293 reference annotations that fell into GO categories for nitrogen, carbohydrate, and phosphorus metabolism. We identified 25 genes with Af293 GO annotations for the term organonitrogen compound metabolic process that were significantly differently abundant between the 3 clades (S12A Fig). Additionally, there were 7 genes with differential abundance for the term carbohydrate metabolic process (S12B Fig) and 5 genes differentially abundant for the term organophosphate biosynthetic process (S12C Fig).

### Distribution of CAZymes

**Fig 6:**
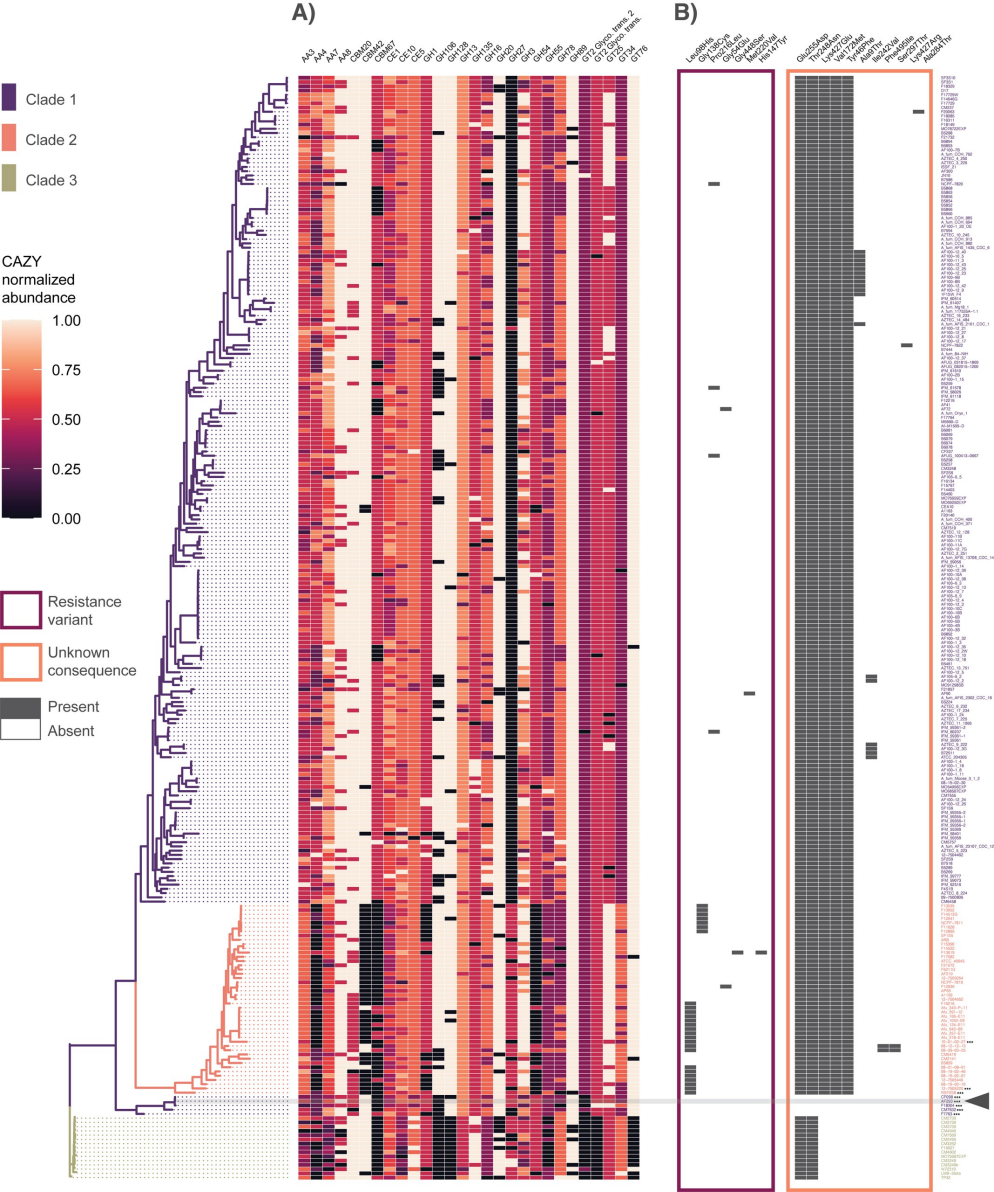
Distribution of CAZymes and occurrence of *cyp51A* mutations across the *A*. *fumigatus* phylogeny. **(A)** CAZyme profiles display patterns of both clade-specific gene gains and clade-specific gene absences across 28 CAZyme classes. CAZyme classes with differential abundance between the 3 clades were determined by Kruskal–Wallis tests at *p* < 0.001 after Bonferroni adjustment for multiple comparisons. Gene counts are normalized on a 0–1 scale (by CAZyme class) for visualization. **(B)** Identification of non-synonymous variants in the *cyp51A* gene across the phylogeny demonstrated structured occurrence of both known resistance variants (framed in pink) and variants with unknown functional impacts (framed in orange). Reference strain Af293 is highlighted in gray and with triangle. While the Leu98His variants (corresponding to the azole resistant TR34/Leu98His genotype) as well as azole resistant variant Gly138Cys were found exclusively in Clade 2, other characterized variants were scattered in low abundance throughout Clade 1, 2, or both. No characterized resistance variants were found in Clade 3. While some non-synonymous variants with unknown functional impacts occurred frequently in *cyp51A* and represented changes in a single branch leading to the reference strain Af293 (Glu255Asp, Thr248Asn), others were absent form this branch and absent in Clade 3 (Lys427Glu, Val172Met, Tyr46Phe), while others were low abundance and exclusive to Clade 1 (Ala9Thr, Lys427Arg, Ile242Val, Ala284Thr), or Clade 2 (Ser297Thr, Phe495Ile). The data underlying this figure can be found in DOI: 10.5281/zenodo.5775265.

To further investigate the role of carbohydrate processing in clade-specific metabolic function, we annotated and assessed the identity and abundance of CAZymes. In total, 139 different CAZyme classes were identified across the *A*. *fumigatus* pan-genome. The CAZyme counts per genome averaged 460 (sd = 7.7, min = 422 in F21732, max = 478 in IFM_59361) (S8 Table). Twenty-eight of these CAZyme classes displayed patterns of differential abundance between the clades (Fig 6A and S9 Table). These abundance profiles represented both copy number variation and clade-specific gene gains and absences in families of auxiliary activities (AAs, *n* = 4), carbohydrate-binding modules (CBMs, *n* = 3), carbohydrate esterases (CEs, *n* = 3), glycoside hydrolases (GHs, *n* = 13), glycosyl transferases (GTs, *n* = 4).

### Presence–absence variance and SNP distribution of antifungal resistance genes and virulence factors

To assess how characterized antifungal resistance genes varied across the phylogeny, we used a combination of reference-based SNP evaluation and de novo gene presence-absence assessment. Across the six genes previously associated with antifungal drug resistance, all were core and present in all isolates—however, the gene family containing *cyp51A* also yielded significant BLAST hits to *cyp51B*, suggesting the possible merger of these closely related genes into a single orthogroup. Because variants were called using reference-based rather than de novo clustering, *cyp51A* and *cyp51B* could however be analyzed separately for the variant analysis. Of the 27 characterized amino acid changing mutations across 6 genes associated with antifungal resistance, we identified functionally characterized variants only in the *cyp51A* gene. In *cyp51A*, a total of 7 resistance variants were identified occurring in a total of 34 strains (Table 1 and Fig 6). The most abundant of these was the TR34/Leu98His genotype (no strains with TR46/Tyr121Phe/Thr289Ala genotype were found). All strains with the TR34/Leu98His genotype, as well as all strains containing the azole resistant variant encoding Gly138Cys, were found exclusively in Clade 2. Other low abundance variants representing characterized *cyp51A*-mediated azole resistance were scattered throughout Clade 1 (Pro216Leu, Met220Val) or Clade 2 (Gly448Ser, His147Tyr), or represented in both Clade 1 and Clade 2 (Gly54Glu). No characterized *cyp51A* resistance variants were found in Clade 3. An additional scan for all non-synonymous *cyp51A* variants revealed 11 additional sites with unknown functional impacts. Two of these sites occurred frequently across the phylogeny and represented changes in a single branch containing Af293 and the other introgressed isolates in Clade 1 (Glu255Asp, Thr248Asn), while 3 were absent from both the Af293 branch and all Clade 3 isolates (Lys427Glu, Val172Met, Tyr46Phe). Six additional sites occurred in low abundance and were either exclusive to Clade 1 (Ala9Thr, Lys427Arg, Ile242Val, Ala284Thr) or Clade 2 (Ser297Thr, Phe495Ile). Other amino acid changing variants in genes associated with resistance, but where the functional consequence of these variants has not been characterized, were found in all other genes investigated, including AFUA7G01960, *artF*, *cdr1B/abcC*, *cox10*, *cyp51B*, *fks1*, *hapE*, *hmg1*, *mdr1*, *mdr2*, *mdr3*, and *mdr4* (S13 Fig). Similarly, these variants of unknown consequence at times represented the dominant allelic state (where the Af293 reference is not representative of the population) and were at other times structured by clade (such as the AFUA7G01960 Leu76Phe variant occurring almost exclusively in Clade 2 or the multiple clade-specific variants in *mdr4* in Clade 3 isolates).

**Table 1:** Occurrence summary of *cyp51A* variants by clade.

	Leu98His	Gly138Cys	Pro216Leu	Gly54Glu	Gly448Ser	Met220Val	His147Tyr
Clade 1	0	0	4	1	0	1	0
Clade 2	19	7	0	1	1	0	1
Clade 3	0	0	0	0	0	0	0
Total	19 (7.25%)	7 (2.67%)	4 (1.52%)	2 (0.76%)	1 (0.38%)	1 (0.38%)	1 (0.38%)

Seven non-synonymous variants associated with antifungal drug resistance were identified in the *cyp51A* gene, representing 34 isolates. Percentages represent the total number of a given amino acid change across all 3 clades out of 261 isolates scanned.

We further used the Af293 gene annotations assigned to gene families to investigate gene presence–absence variation across 26 notable *A*. *fumigatus* secondary metabolite biosynthetic gene clusters (BGC, as defined in [112]) (S14 Fig). Out of the 230 genes investigated, 7 were convoluted with one other gene in the same cluster, where both genes clustered into the same gene family. These included Afu3g13700/Afu3g13690 in BGC 10, Afu4g00220/Afu4g00210 in BGC 13, Afu4g14560/Afu4g14550 in BGC 14, Afu6g09730/Afu6g09720 in BGC 20, Afu6g13980/Afu6g13970 in BGC 22, Afu8g00490 Afu8g00480 in BGC 25, and Afu8g02380/Afu8g02360 in BGC 26. Three genes (Afu1g10275 in BGC 2, Afu7g00140 in BGC 23, and Afu8g00450 in BGC 25) could not be confidently assigned to a gene family. We found substantial variation in conservation across the 26 clusters, with the lowest conservation in BGC 1 (Uncharacterized polyketide—with 259 strains missing at least 1 gene), BGC 12 (Uncharacterized non-ribosomal peptide, 240 strains missing at least 1 gene), BGC 16 (Uncharacterized non-ribosomal peptide-like, 134 strains missing at least 1 gene), and BGC 13 (Endocrocin, 112 strains missing at least 1 gene). Additionally, Afu8g00400 in cluster 25 could only confidently be called in 1 isolate (AF100-1_24). Eleven BGCs demonstrated high levels of conservation, with less than 10 strains missing any gene in the cluster. The most conserved was BGC 24 (Fumitremorgin) for which all genes were present in all strains, followed by BGC 8 (Fusarine C) with genes missing in only 3 strains, BGC 11 (Uncharacterized polyketide) with genes missing in only 4 strains, BGCs 21 (Fumiquinozalines) and 3 (Ferricrocin), with genes missing in only 5 strains, BGC 7 (Uncharacterized polyketide) missing genes in only 6 strains, BGCs 6 (Fumigaclavine), 9 (Hexadehydroastechrome), and 19 (Uncharacterized non-ribosomal peptide), all missing in only 7 strains, BGC 5 (DHN Melanin) missing in only 8 strains, and BGC 23 (Neosartoricin) missing in 9 strains. We further investigated the likely implications of gene loss in the 13 genes encoding the gliotoxin cluster (BGC 20) across the *A*. *fumigatus* phylogeny. These 13 genes were assigned to 12 gene families (with *gliF* and *gliN* both assigned to the same family). All 12 were highly conserved with low levels of gene absences. An exception to this finding was the wholesale loss of *gliI*, *gliJ*, and *gliZ* from 8 strains clustered in Clade 1 (S14 Fig). All strains represent isolates from a long-term infection of a cystic fibrosis patient [119]. Because *gliI*, *gliJ*, and *gliZ* are immediately adjacent in the Af293 reference genome, we further investigated the boundaries of this deletion and found that it encompasses a total 18,261 BP, spanning Chr6 2336977..2355238 in Af293 reference coordinates. This deletion included 454 BP of the gene Afu6g095906, whole gene losses of the genes Afu6g09600, Afu6g0910, Afu6g09620, Afu6g09630 (*gliZ*), Afu6g09640 (*gliI*), Afu6g09650 (*gliJ*), and 2619 BP of Afu6g09660 (*gliP*) (S16B Fig).

### Spatial enrichment

To identify differential spatial distribution in core, accessory, and singletons gene families across each genome, we performed enrichment analysis of each gene category relative to the distance to chromosome ends. We identified significant depletion of core gene families (W = 51,142,943, *p* < 0.001, S15A and S15B Fig), and significant enrichment of both accessory (W = 16,070,357, *p* < 0.001, S15C and S15D Fig) and singleton (W = 3850, *p* < 0.001, S15E and S15F Fig) gene families within 50 kb of telomeres.

## Discussion

### Population structure and recombination rate

Due to the high dispersibility and assumed substrate generalism, *A*. *fumigatus* was originally thought to represent a single homogenous population [28,29]. However, recent investigations have found mixed evidence for both population stratification and recombination frequency [30,32,33]. Importantly, tests for recombination that do not take population structure into account, or incorrectly infer the number of underlying populations, can bias estimates of recombination rate [120]. Contrary to models of panmixia, we found strong evidence for 3 distinct populations of *A*. *fumigatus*, with secondary population structure in both Clade 1 and Clade 2 (Figs 1A and S3). We identified only 8 strains with mixed ancestry between the 3 primary clades (Fig 1B and 1C). These included 3 strains assigned to Clade 2 and 5 strains assigned to Clade 1—including the Af293 reference strain. All 5 strains with mixed ancestry assigned to Clade 1 clustered together on the same branch that was situated apart from the rest of Clade 1 and between Clades 2 and 3, an arrangement typical of mixed ancestry (Fig 4). As shared genetic variation between clades could be indicative of either the retention of ancestral polymorphisms associated with incomplete lineage sorting during speciation or introgression (hybridization) events between clades, we attempted to distinguish between these 2 scenarios using TreeMix. We found that the best migration model supported gene flow from Clade 3 to the 5 introgressed isolates in Clade 1 (S4 Fig). Similarly, Patterson’s D supported introgression over incomplete lineage sorting. Taken together, these results indicate that the branch containing Af293 is the result of a hybridization event, rather than a speciation event, and substantiate its membership in Clade 1 over elevating this branch to a fourth clade. Overall, we found that genetic differentiation was lower between Clades 1 and 2, with Clade 3 more divergent. While Clade 3 contained no population substructure, Clades 1 and 2 each contained approximately 5 sub-clades (S3D and S3E Fig). Interestingly, while evidence for introgression between the 3 primary clades was limited to a few strains, within-clade introgression appeared to be common (S3F and S3G Fig). To investigate further, we quantified recombination frequency using LD decay (a pairwise site comparison of the decrease in LD across genome space) and found that LD50 was startlingly low (1.97 BP) when assessed across all isolates (Fig 1D–1G). Because LD estimates can be biased by ignoring population structure, we also conducted LD assessments for each clade individually and overall for different values of n-samples. We found that sample-size normalization greatly increased the overall LD estimates (from 1.97 BP to 308.08 BP across all isolates), and that while LD varied for each clade (lowest for Clade 1 and highest in Clade 3), LD values remained extremely low, indicating exceptionally high levels of recombination in *A*. *fumigatus*. For comparison, *Candida albicans* (considered to be obligately asexual) has a reported LD50 of 162,100 [121]. Conversely, *A*. *flavus*, which demonstrates relatively frequent outcrossing, has an LD50 value estimated between 1,000 to 12,300 [122]. The exceptional level of recombination identified here brings up important questions about the mechanisms facilitating the generation of genetic diversity in organisms that disperse using primarily asexual means. Recent estimates of recombination rate have singled out *A*. *fumigatus* as potentially having a higher number of crossovers per meiotic event than any eukaryotic species investigated to date [45]. Extremely high recombination rates would help explain the extremely low LD decay values and exceptionally large pan-genome size observed in *A*. *fumigatus*. Given that LD estimation is often carried out without regard to population parameters or sample size, these results also highlight the sensitivity of LD estimates to cofounding variables and the importance of population structure and normalization in the interpretation of LD decay.

Although sexual recombination has been documented to occur in *A*. *fumigatus* [36], the frequency of these recombination events in natural populations is unknown. One indicator of sexual recombination is the population frequency of MAT-type idiomorphs, where ratios close to 1:1 are indicative of random mating [123]. We found MAT1-1:MAT1-2 ratios in of approximately 7:5, with both mating types present in each primary clade, congruent with ongoing sexual recombination in natural populations. Unexpectedly, we identified 11 strains with both mating type idiomorphs. To further investigate these strains, and the potential for low levels of diploidy in *A*. *fumigatus*, we first analyzed the read depth profiles of the full-length alignments to both MAT1-1 and MAT1-2. We found that within a single strain, each idiomorph displayed differing sequencing depth profiles in all but 3 cases (AF100-1_3, IFM_59359, and IFM_61407) (S9 Fig), suggesting the potential for different underlying causes of the double mapping to both MAT loci in these strains. Interestingly, one of these strains, Afu_343_P_11, was previously identified as an outlier [30], for its exceptional genetic diversity. Similarly, K-mer ploidy analysis supported haploidy in 7 of the 9 strains, but diploidy for AF100-1_3 and IFM_59359 and allele frequency analysis was in agreement with the depth analysis, supporting diploidy for AF100-1_3, IFM_59359, and IFM_61407, and haploidy for all others. Taken together, while at least 6 of the 9 strains with double MAT alignments may represent either partial duplications, or low levels of noise (culture contamination or tag switching during the sequence process), others provide evidence for low levels of diploidy in the *A*. *fumigatus* population. The diploid state is a signature of parasexual recombination and low incidence of *A*. *fumigatus* diploid strains have been previously found in *A*. *fumigatus* lung isolates from cystic fibrosis patients [124]. While AF100-1_3 is a clinical cystic fibrosis lung isolate [119], IFM_59359 is a clinical isolate taken from a patient with pulmonary aspergilloma, and IFM_61407 is a clinical isolate from a patient with chronic necrotizing pulmonary aspergillosis [125]. Thus, all 3 of these strains had the potential to be in the human lung environment for a long period of time. Overall, the impact and relative frequency of sexual versus parasexual recombination in *A*. *fumigatus* is unclear and a critical topic for future research.

### Pan-genome diversity

The *A*. *fumigatus* pan-genome contained 5,180 more gene families than there are genes currently predicted in the Af293 reference genome (15,309 identified here versus 9,840 protein coding genes, and 289 noncoding RNA genes currently predicted in Af293 in FungiDB as of October 2021). Because gene families represent clusters of homologues, including both orthologues and paralogues, genes and gene families are not expected to correlate in a one-to-one ratio, and the number of true genes in the *A*. *fumigatus* pan-genome is likely even higher than the number of gene families reported here. Method validation for our pipeline for de novo assembly and pan-genomic clustering identified 10,167 genes (in 10,005 gene families) in the re-sequenced reference strain AF293, 38 more than are currently predicted in the curated Af293 reference strain. The small discrepancy between the number of genes identified in the pan-genomic analysis over those currently annotated in the reference highlights the high fidelity of the methods used here, where the additional genes identified for AF293 may represent either low levels of error in gene calling, or genuine variation between the reference Af293 isolate and the re-sequenced “AF293” isolate [126].

While the majority of gene families identified were part of the core genome of *A*. *fumigatus* (57.9%) (Fig 2A), substantial numbers of gene families were identified as accessory (28.3%) or singleton genes (13.8%). While the total abundance of singleton and accessory gene families were not structured by phylogeny or enriched by clade (Fig 3), we found that accessory genes were more evenly distributed than singletons and that high average numbers of singletons were driven by a relatively small number of strains with high gene diversity (Fig 2B–2D). The high number of genes appearing in only a single isolate might suggests that these genes are more likely to be error prone or less likely to contain functional information. However, we were able to assign functional annotations to the vast majority of both accessory (79.48%) and singleton (81.27%) gene families, suggesting that singletons are likely functional and not artifacts of the pipeline. Similarly, although singleton gene families may represent overly stringent-binning of accessory gene families, the similar prevalence of singleton gene families between OrthoFinder and PIRATE defined homologues, leads us to conclude that these families represent substantial genetic diversity in gene families that occur only rarely in the greater population. Given that core gene families are considerably more likely to be present in reference strains, and therefore more likely to be characterized, the high annotation frequency in core genes (98.25%) is to be expected. The absence of functional annotation for approximately 20% of the dispensable genome highlights the shortcomings of single reference-based approaches for investigating population-wide genetic diversity.

Despite our ability to assign functional annotations to the majority of the dispensable genome, the distribution of accessory and singletons gene families should be considered separately. Gene accumulation curves demonstrated a closed pan-genome structure when considering only accessory gene families, but an open pan-genome structure with unsaturated genetic diversity when singletons were included (Fig 2C). The diversity of singleton genes in *A*. *fumigatus* suggests both a mechanism for the generation of substantial genetic diversity, and a mechanism for the purging of much of this diversity before these novel singleton genes become fixed in the population.

Our results suggest that *A*. *fumigatus* has a pan-genome size of approximately 67.2:32.8 core:accessory gene families excluding singletons and 57.9:42.1 core:accessory gene families including singletons. This ratio signifies an exceptionally large dispensable genetic repertoire and represents one of the largest fungal pan-genomes ever reported. Pan-genome size is influenced by population size, outcrossing frequency, and niche specificity [21,43], where species with large populations, frequent gene flow, and substrate heterogeneity are associated with larger pan-genomes. For example, *Zymoseptoria tritici* is a highly outcrossing, widely distributed wheat pathogen with an estimated ratio of 60:40 core:accessory [127]. For comparison, *Saccharomyces cerevisiae* is also widely distributed, but rarely outcrossing, and has a ratio of 93.4:6.6 core:accessory genes [128]. Previous pan-genomic analysis of *A*. *fumigatus* have found ratios of core:accessory genes ranging from 83.29:16.71 core:accessory using 12 isolates [129] to 69:31 core:accessory using 300 isolates [31]. Ecologically, the exceptional diversity observed in the pan-genome of *A*. *fumigatus* is likely driven by the massive population size and global distribution of the species, coupled to environmental heterogeneity, and possibly by cryptic adaptation and yet undiscovered niche specificity.

Signatures of clade-specific genetic diversity were evident in all 3 primary clades, with each containing numerous clade-specific gene families (Fig 3A). While the total number of unique gene families in each clade was intrinsically influenced by differences in clade size, we can generalize about the distribution of these gene families within clades. For example, while many clade-specific gene families were present in only a minority of isolates, others were present in all or nearly all of the isolates in a given clade. Presence–absence analysis also identified gene families that were uniquely absent among a single clade (Fig 3B), including multiple clade-defining absences, where gene families were missing in all isolates of that clade, but present in nearly all isolates of the other 2 clades. Here, absence could represent either gene loss in the clade of interest or gene gains in the other 2 clades. Overall, fewer absences were identified in Clade 1 than for the other 2 clades, with no gene families making our cutoff to define the loss as a clade-defining feature. Conversely, clade-defining absences were present in both Clade 2 (*n* = 2 absences) and Clade 3 (*n* = 23 absences).

Although the mechanisms facilitating the remarkable genetic diversity observed in *A*. *fumigatus* are unknown, the generation of novel dispensable genes may be associated with genomic region, such as distance to chromosome ends [15]. Subtelomeric regions undergo rapid expansion and contraction events and increased rates of evolution and recombination during both meiosis and mitosis [18,130,131], generating genomic diversity that may particularly impact genes needed for rapid adaptation such as those involved in pathogenicity [5,19] and metabolism [18]. In accordance, we found significant enrichment of both singleton and accessory genes in subtelomeric regions, along with significant depletion of core genes in these regions (S15 Fig). Despite strong enrichment for dispensable genes at chromosome ends relative to internal regions, it should be noted that the majority of dispensable genes were found distal to telomers. This dichotomy suggests a possible mechanism for the generation of genomic diversity in subtelomeric regions, followed by the subsequent translocation of these genes to regions internal to chromosomes, a model that has been proposed for the generation of metabolic diversity in *Cryptococcus* [132].

### Functional enrichment

Whereas core gene families were enriched for GO terms related to expected essential functions such as transcription, translation, and transport, we found that the dispensable genome was enriched for terms related to metabolism—particularly terms related to nitrogen, carbohydrate, and phosphorus processing (Fig 5A–5C). Enrichment of clade-specific gene families echoed these results, with various metabolic processes enriched in clade-specific gene families in Clades 1 and 3 (Fig 5D and 5E). To further investigate the potential for differential metabolic capacity across the phylogeny, we first annotated CAZyme encoding genes across all isolates and identified clade-wise differential abundance in 28 CAZyme families (Fig 6A). These CAZymes represent diverse metabolic activities including carbohydrate-binding modules, carbohydrate esterases, glycoside hydrolases, glycosyl transferases, and auxiliary activities. These gene families are associated with diverse roles in colonization, nutrient utilization, and substrate specificity [133]. We further looked for evidence of substrate specificity using gene family homology to Af293 annotations to identify clade-specific patterns in gene presence–absence variation in genes associated with nitrogen, carbohydrate, and phosphorus metabolism. We found evidence for significant clade-wise presence–absence variation in all 3 gene categories (S12A–S12C Fig). For example, Afu6g14620, which has been largely lost in Clade 2, but not Clades 1 and 3, encodes a putative Alpha-L-arabinofuranosidase, an enzyme that hydrolyzes arabinose side chains, and is known to be substrate specific [134].

Clade-specific differences in genes involved in primary metabolism are likely driven by ecological and evolutionary factors relevant to environmental systems, such as niche occupation and subtle substrate specificity on various plant biomass materials. However, genetic differences driven by environmental ecological factors may also have clinical implications. For example, GH135 (*sph3*, with an additional copy in Clade 3) is involved in the production of galactosaminogalactan (GAG), a critical component of *A*. *fumigatus* biofilms [135]. GH16 and GH55 (both with 1 less copy in Clade 3) are essential for proper *A*. *fumigatus* conidial morphogenesis [136,137]. AA3 (with an additional copy in Clade 2, and AA7 (with 1 less copy in Clade 2, and 2 less copies in Clade 3) likely serve as oxidases with a role in the production of H_2_O_2_ for lignin depolymerization [138]. However, differences in the production of H_2_O_2_ may require corresponding differences in detoxification ability and could impact interactions with host leukocytes that employ oxidative antifungal killing mechanisms [139–143]. Metabolic specificity may also represent the possibility for niche preadaptation in clinical settings, as nutritional landscapes may not be uniform in clinical environments. For example, the cystic fibrosis lung environment is characterized by the impaired clearance and increased viscosity of mucins [144]. These mucins represent unique carbon and nitrogen sources that act as substrates for diverse microbial communities [145] with the potential for both direct and syntrophic interactions between colonizing fungi and bacteria.

Genes involved in secondary metabolism may also underlie clade-specific niche occupation, as the expression of biosynthetic clusters is tied to differential nutrient access and nutrient sensing [146]. Here, we found substantial presence–absence variation in BGCs encoding diverse secondary metabolites (S14 Fig), and these too may have human disease implications. For example, the genes Afu3g13730, Afu3g13720, and Afu3g13710, which are lost in Clade 3 but present in nearly all other isolates, are part of the same uncharacterized biosynthetic NRPS-like gene cluster [112]. Also coded under the GO terms for organonitrogen and organophosphorus metabolism, this cluster is preferentially expressed during the initial 4 h of infection in-vivo [112].

Another example with potential impacts on virulence and pathogenesis is the discovery of isolates lacking part of the gliotoxin BGC (S14 Fig). Gliotoxin is a powerful mycotoxin and virulence factor in *A*. *fumigatus* infection [147]. A large deletion in 8 strains, covering 8 genes on Chromosome 6 (relative to Af293), included the tailoring and structural genes *gliI* and *gliJ*, the transcription factor *gliZ*, and a partial deletion in the NRPS gene *gliP*. Among these, *gliI* and *gliP* are considered essential to gliotoxin biosynthesis [148,149]. Whereas the full *gliP* protein contains 2 sets of canonical NRPS A-T-C modules plus a tailing T (thioesterase), the truncated gene is predicted to encode a single A-T-C module and a tailing A, making peptide formation unlikely (S16 Fig). As the production of gliotoxin is thought to impart enhanced survival and infection persistence [150], the presence of this large deletion in 8 strains representing a persistent lineage present in a cystic fibrosis patient with long-term aspergillosis [119] hints that gliotoxin production is not necessary for persistence in patients with cystic fibrosis and highlights possible alternative/adaptive roles for the functional loss of this cluster under some conditions. Although the results presented here identify an intriguing direction for future study, functional work is needed to clarify realized differences in substrate usage and any potential links between ecological niche occupation and realized differences in pathogenesis and virulence traits.

### Antifungal resistance genes

To test if clinically relevant alleles were also biased in their distribution across the 3 clades, we conducted variant scans on a set of 13 genes associated with antifungal drug resistance. These included targeted analysis of 6 functionally characterized amino acid changes available for 6 genes (*cyp51A*, *cyp51B*, *hapE*, *hmg1*, *cox10*, and *fks1*) (S2 Table), as well as surveying all amino acid changing variants across these 6 genes and an additional 7 genes associated with azole resistance, but lacking functional characterization of specific amino acid changes. Targeted scans only identified previously characterized mutations only in the *cyp51A* gene (Fig 6B). While the azole resistant TR34/Leu98His and Gly138Cys genotypes were found exclusively in Clade 2, other characterized variants in *cyp51A*-mediated azole resistance were scattered in low abundance throughout Clade 1 (Pro216Leu, Met220Val) or Clade 2 (Gly448Ser, His147Tyr), or represented in both Clade 1 and Clade 2 (Gly54Glu). Conversely, no characterized *cyp51A* resistance variants were found in Clade 3. An additional scan for all non-synonymous variants in *cyp51A* found 11 amino acid changes that occurred frequently across the phylogeny. Interestingly, 2 of these changes (Glu255Asp and Thr248Asn) are variants that map to a monophyletic branch containing the Af293 reference Clade 1 isolates with signs of introgression, making the Glu255Asp and Thr248Asn genotypes the rule rather than the exception and highlighting the potential problems associated with defining mutations relative to a single reference. Similarly, 3 variants (Lys427Glu, Val172Met, and Tyr46Phe) were absent only from the monophyletic branch containing the reference and from all Clade 3 isolates. Finally, 6 additional variants were present only in low abundance and exclusive to Clade 1 (Ala9Thr, Lys427Arg, Ile242Val, Ala284Thr) or Clade 2 (Ser297Thr, Phe495Ile). While the functional impacts of these low abundance amino acid changes are unknown, the frequency of uncharacterized *cyp51A* amino acid changes deserves future consideration. Additionally, while the genes scanned did not contain characterized drug resistance alleles, they all contained multiple uncharacterized amino acid changing variants (S13 Fig). Several of these changes demonstrated phylogenetic structure, such as the Leu76Phe change in AFUA7G01960, which was both prevalent and nearly exclusive to Clade 2 isolates. As *cyp51A* mutations in *A*. *fumigatus* only account for an estimated 43% of resistant isolates [41], the distribution of non-synonymous variants in other genes associated with resistance highlight the importance of future research on the functional impact and population distribution of alternative drug resistance mechanisms in *A*. *fumigatus*.

## Conclusion

The availability and continued improvement of high-quality reference genomes has enabled insights into the evolution and variation of fungal genome structure [151], intragenomic evolutionary rates [152,153], and how intraspecific genetic variability is structured across fungal populations [154,155]. However, there is growing appreciation that a single reference genome is incapable of capturing the genetic variation present across a species. Whereas genes absent in the reference genome are likely to be ignored using reference-based alignment, de novo strategies for genome assembly offer the possibility of capturing the full repertoire of genetic diversity [156]. Here, we used a combined genomics approach to leverage both reference-based and de novo strategies to illuminate population structure, recombination frequency, and genetic diversity across the *A*. *fumigatus* pan-genome. These populations, subdivided into 3 primary clades, are characterized by exceptionally high gene diversity and substantial presence–absence variation, representing one of the largest fungal pan-genomes ever reported. Our results suggest that recombination occurs at strikingly high rates in *A*. *fumigatus*, but that the frequency of these recombination events happens primarily within-clade and only rarely between clades. Laboratory studies are needed to confirm the recombination rate, frequency of mating, and mating compatibility within and between isolates from the 3 primary lineages. We found that the 3 primary clades are defined by genes encoding diverse metabolic functions, hinting that population structure may be shaped by environmental niche occupation or substrate specificity. If niche occupation translates to realized differences in nutrient usage or stress tolerance, it may have implications for disease initiation and/or progression [35]. Finally, as evidenced by the numerous gene families identified here which have no homologue in Af293, the under-characterization and inability to assign annotations to approximately 20% of the dispensable genome, and variant profiles that identified clear cases where Af293 was the exception, rather than the rule for the population (and therefore inappropriate to define mutations against), this work highlights the inadequacy of using single reference-based approaches to capture the genetic variation across a species, and the power of combined genomics approaches to elucidate intraspecific diversity. As this study can only represent conclusions based on the data available, we anticipate that as additional genome sequences become available, novel genomic and phylogenetic diversity will continue to be discovered in *A*. *fumigatus*. Additionally, the methodological hurdles associated with assessing population structure and recombination frequency in organisms with complex clonal-sexual life cycles is significant and highlights the need for the further development of computational tools capable of addressing issues specific to fungi.

## Supporting information

S1 Fig fastSTRUCTURE marginal likelihood values. Marginal likelihood increased until K = 5, with the largest increase occurring between K = 1–3. The data underlying this figure can be found in DOI: 10.5281/zenodo.5775265.
(TIF)

S2 Fig Statistical standards for *A*. *fumigatus* population structure. **(A)** BIC score vs. number of clusters, with elbow of the curve at K = 3 clusters, **(B)** choice of PCs for DAPCA was found to be optimal at PC = 3 according to a-score. The data underlying this figure can be found in DOI: 10.5281/zenodo.5775265. BIC, Bayesian information criterion; DAPC, discriminate analysis of principle component.
(TIF)

S3 Fig Investigation of population substructure. Population substructure within the 3 clades was investigated using iterative DAPC and fastSTRUCTURE analysis of vcf SNP sites subset by clade. **(A)** The optimal number of PCs for the sub-clades was determined to be 5 for the substructure within Clade 1. **(B)** The optimal number of PCs for the sub-clades was determined to be 3 for the substructure within Clade 2. **(C)** The optimal number of PCs for Clade 3 was determined to be 1 and was therefore not further analyzed with DAPC. **(D)** Iterative DAPC analysis supported 5 sub-clades within Clade 1. **(E)** Iterative DAPC analysis supported 5 sub-clades within Clade 2. **(F)** Iterative fastSTRUCTURE at K = 5 supported frequent introgression between sub-clades in Clade 1 (87 out of 200 strains). **(G)** Iterative fastSTRUCTURE at K = 5 supported frequent introgression between sub-clades in Clade 1 (7 out of 45 strains). Sub-clade designations were not consistent between DAPC and fastSTRCTURE (i.e., DAPC sub-clade 1.1 is not equivalent to fastSTRUCTURE sub-clade 1.1). The data underlying this figure can be found in DOI: 10.5281/zenodo.5775265.
(TIF)

S4 Fig TreeMix results. TreeMix was run on a VCF file containing all strains plus *A*. *fischeri* as the outgroup with the optimal number of migration edges determined using the program OptM, using 10 independent iterations of m ranging from 1–10. **(A)** Variance explained across different values of m and **(B)** delta m spike supporting the optimal migrations at m = 1. **(C)** TreeMix graph supporting gene flow from Clade 3 to sub-clade 1.1, containing 5 introgressed isolates with majority ancestry in Clade 1 and including the reference strain Af293. The data underlying this figure can be found in DOI: 10.5281/zenodo.5775265.
(TIF)

S5 Fig The influence of sample size on LD decay. To investigate the influence of sample size on LD decay, we iteratively sampled *n* = 5, 10, 20, 50, or 100 isolates without regard to population structure (each *n* averaged over 20 independent iterations) and calculated LD50 in BPs. The influence of sample size on LD estimates showed a strong inverse relationship between sample size and LD decay, with an LD50 of 1,489.55 BP at *n* = 5, 308.80 BP at *n* = 10, 5.02 BP at *n* = 20, 4.7 BP at *n* = 50, and 2.76 BP at *n* = 100. **(A)** Linear–linear plot. **(B)** Zoomed in log-linear plot, with arrows denoting LD50 at each *n*. The data underlying this figure can be found in DOI: 10.5281/zenodo.5775265. BP, base pair; LD, linkage disequilibrium.
(TIF)

S6 Fig Pan-genome gene family distribution using PIRATE. **(A)** The pan-genome of 260 *A*. *fumigatus* strains included 15,476 gene families in total, including 8,600 (55.57%) core genes (present in >95% of strains), 3,618 (13.92%) accessory genes (present in >2 and <248 strains), and 3,258 (21.05%) singletons (present in only 1 isolate). **(B)** The distribution of the number of genomes represented in each gene family. **(C)** Gene family accumulation curves, including (green) and excluding (yellow) singletons. **(D)** The distribution of unique accessory gene families by strain. **(E)** The distribution of unique singleton gene families by strain. The data underlying this figure can be found in DOI: 10.5281/zenodo.5775265.
(TIF)

S7 Fig Relationship between genome size and number of gene families. Both **(A)** accessory gene families and **(B)** singleton gene families were significantly related to predicted genome size, although this relationship yielded low R^2^ values, particularly for singleton gene families. The data underling this figure can be found in https://github.com/MycoPunk/Afum_PopPan.
(TIF)

S8 Fig Proportion of secreted proteins across the pan-genome by frequency and clade. Secreted proteins were predicted using the programs Signal P and Phobius. In most cases, Phobius predicted a slightly greater number of secreted proteins than Signal P. Both Signal P (lightest shading) and Phobius (mid-level shading) contained proteins not annotated by the other program, but most individual proteins called as secreted overlapped between the 2 prediction programs (darkest shading). **(A)** Proportion of gene families predicted to represent secreted proteins out of all gene families by frequency (core, accessory, and singleton) and **(B)** by clade for clade-specific gene families. In Clade 3, only 2 categories are depicted as all secreted proteins predicted by Signal P were also predicted by Phobius. Significant differences were assessed using Pairwise proportion tests with Bonferroni adjustment for multiple comparisons at *p* < 0.05. Letters (lowercase) in common, indicate no significant difference between groups. Significance results were consistent across Signal P, Phobius, and Consensus inferred secreted proteins. The data underlying this figure can be found in DOI: 10.5281/zenodo.5775265.
(TIF)

S9 Fig Depth profiles of MAT idiomorph alignments for the 9 isolates with significant alignments to both MAT1-1 and MAT1-2, plus positive controls. All strains had alignments over the entirety of the MAT region for both idiomorphs, but these alignments different in depth, suggesting different underlying explanations. For MAT1-2 alignments, the trailing approximately 270 BP region that is conserved between MAT1-1 and MAT1-2 is visible here in the control as well as in several of the isolates that mapped to both idiomorphs. While MAT1-2 idiomorph strains have a full alignment over the (approximately 1,078 BP) MAT1-2 reference, MAT1-1 idiomorph strains only align over the approximately 270 BP conserved region of the MAT1-2 reference. The data underlying this figure can be found in DOI: 10.5281/zenodo.5775265.
(TIF)

S10 Fig K-mer profiles of strains with alignments to both MAT1-1 and MAT1-2 while 7 of the 9 strains display single peaks indicative of haploidy, 2 strains, AF100-1_3 and IFM_59359 display low secondary peaks, potentially indicative of diploidy. The data underlying this figure can be found in DOI: 10.5281/zenodo.5775265.
(TIF)

S11 Fig Allele frequency profiles of strains with alignments to both MAT1-1 and MAT-2 plots indicate genome-wide allelic ratios across all SNPs for each genome, where 1 or 0 indicates homozygosity (1 indicating a polymorphism and 0 matching the reference allele of Af293). Although homozygous positions are expected to dominate the data (outer graph), diploids are expected to have an additional peak at an allele frequency of 1/2, visible when 1 and 0 are removed from the graph space (inner plot). While 6 of the 9 strains display no notable peaks at 1/2, 3 strains (AF100-1_3, IFM_59359, and IFM_61407) do show peaks at 1/2. Four other strains, while likely haploid, display significant areas of copy number variation as evidenced by additional peaks just below 1/4 and just above 3/4. The data underlying this figure can be found in DOI: 10.5281/zenodo.5775265.
(TIF)

S12 Fig Presence–absence variation in metabolic genes. Gene family clusters with significant homology to annotated genes the Af293 reference genome were assigned using BLASTP (e-value < 1e^-15^). Using these annotated clusters, we identified differential abundance in clusters with Af293 annotations between the 3 clades, for Af293 genes in FungiDB under the GO terms **(A)** organonitrogen compound metabolic process (GO: 1901564, *n* = 25 significantly differentially abundant genes; only the first 7 are depicted for visualization), **(B)** carbohydrate metabolic process (GO: 0005975, 7 significantly differentially abundant genes), and **(C)** organophosphate biosynthetic process (GO: 0090407, 5 significantly differentially abundant genes). Genes denoted with * are shown grouped with GO: 1901564, but are also annotated in GO: 0090407. The data underlying this figure can be found in DOI: 10.5281/zenodo.5775265.
(TIF)

S13 Fig Distribution of non-synonymous variants in non-*cyp51A* genes associated with fungal drug resistance. In addition of *cyp51A*, a database of other genes known to be associated with antifungal drug resistance was assembled (S2 Table), and all isolates were scanned for non-synonymous variants in these genes. Four genes, Cyp51b, cox10, mdr2, and AFUA 7G01960 contained uncharacterized non-synonymous amino acid changes, several of which demonstrated phylogenetic structure. The data underlying this figure can be found in DOI: 10.5281/zenodo.5775265.
(TIF)

S14 Fig Presence–absence variation in secondary metabolite biosynthetic gene clusters. Gene family clusters with significant homology to annotated genes the Af293 reference genome were assigned using BLASTP (e-value < 1e^-15^) and genes in gene clusters encoding notable *A*. *fumigatus* secondary metabolites (as defined in [112]) were mapped onto the phylogeny in R. Out of 230 genes in 26 clusters, 7 were convoluted with one other gene in the cluster, where both genes clustered into the same gene family (in these cases, both genes are listed separated by /). Three genes (Afu1g10275 in cluster 2, Afu7g00140 in cluster 23, and Afu8g00450 in cluster 25) could not be confidently assigned to an Orthofinder gene family and are not displayed. The data underlying this figure can be found in DOI: 10.5281/zenodo.5775265. BGC, biosynthetic gene cluster.
(TIF)

S15 Fig Enrichment of gene families relative to telomeres. The relative abundance of core, accessory, and singleton gene families was calculated over 50 kb windows for all contigs >50 kb in length and containing a telomeric repeat. Possible enrichment of each abundance category was then tested using a 2-sided Wilcoxon rank sum test implemented in the *stats* package in R, showing significant depletion of core genes and significant enrichment of singleton and accessory genes within 50 kb of telomere ends. The data underlying this figure can be found in DOI: 10.5281/zenodo.5775265.
(TIF)

S16 Fig Presence–absence variation in gliotoxin cluster genes. The boundaries of the deletion were the same for all 8 strains and included the partial truncation of gliP, covering 1 A-T-C module. The data underlying this figure can be found in DOI: 10.5281/zenodo.5775265.
(TIF)

S1 Table Accession numbers and strain information. (XLSX)

S2 Table Database of characterized antifungal resistance mutations in *A*. *fumigatus*. Database was assembled from the literature and supplemented with search results from the MOADy antifungal drug resistance database.
(XLSX)

S3 Table DAPCA clade assignments, including posterior probabilities for each strain used in this study. (XLSX)

S4 Table fastSTRUCTRE clade assignments, including posterior probabilities for each strain used in this study. (XLSX)

S5 Table DAPC sub-clade assignments, including posterior probabilities for each strain. (XLSX)

S6 Table fastSTRUCTURE sub-clade assignments, including posterior probabilities for each strain. (XLSX)

S7 Table Abundance and annotation of clade-specific gene families. (XLSX)

S8 Table CAZyme gene counts for all *A*. *fumigatus* isolates. (XLSX)

S9 Table CAZyme enrichment stats table. Mean gene counts and post-hoc results for the 28 CAZymes with significantly different abundance between the 3 clades. Letters (in parenthesis) in common indicate no significant difference in abundance.
(XLSX)

## Abbreviations:

AA: auxiliary activity
AAFTF: Automatic Assembly For The Fungi
AMOVA: analysis of molecular variance
BGC: biosynthetic gene cluster
BIC: Bayesian information criterion
BP: base pair
CBM: carbohydrate-binding module
CE: carbohydrate esterase
DAPC: Discriminant Analysis of Principal Components
GA: galactosaminogalactan
GH: glycoside hydrolase
GO: Gene Ontology
GT: glycosyl transferase
HMG: high-mobility group
LD: linkage disequilibrium
PIRATE: Pangenome Iterative Refinement and Threshold Evaluation
TE: transposable element
