# Supplementary figures and images for: The pan-genome of Aspergillus fumigatus provides a high-resolution view of its population structure revealing high levels of lineage-specific diversity driven by recombination
Source: PLoS Biol. 2022 Nov 17;20(11):e3001890. doi: 10.1371/journal.pbio.3001890 (PMC9714929; doi:10.1371/journal.pbio.3001890)

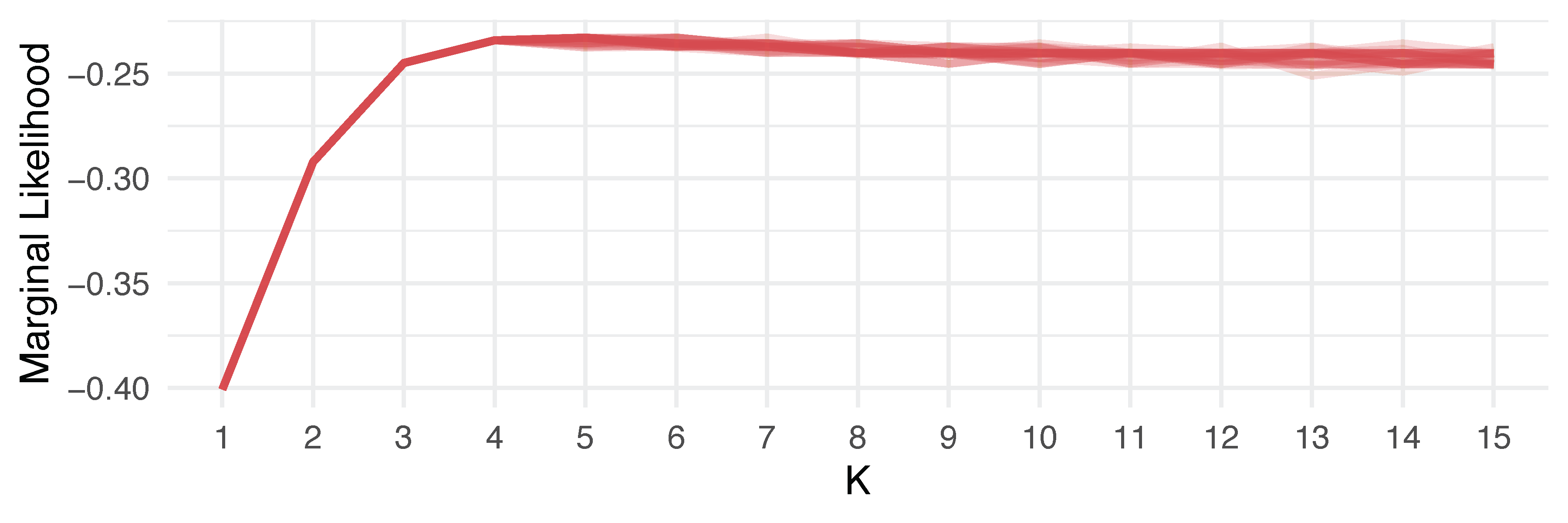

Supplement: S1 Fig — Marginal likelihood increased until K = 5, with the largest increase occurring between K = 1–3. The data underlying this figure can be found in DOI: 10.5281/zenodo.5775265. (TIF) [file pbio.3001890.s001.tif]

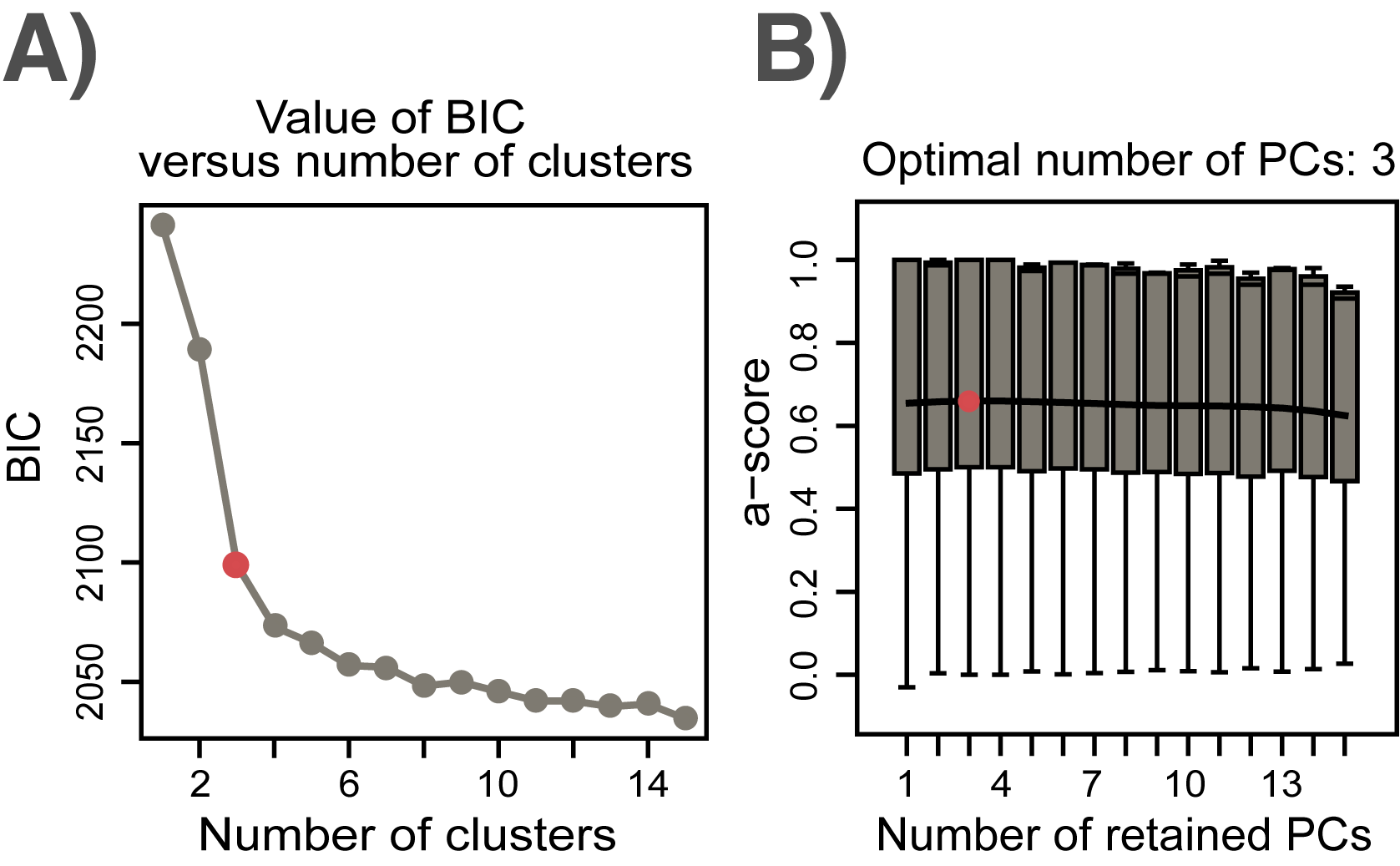

Supplement: S2 Fig — (A) BIC score vs. number of clusters, with elbow of the curve at K = 3 clusters, (B) choice of PCs for DAPCA was found to be optimal at PC = 3 according to a-score. The data underlying this figure can be found in DOI: 10.5281/zenodo.5775265. BIC, Bayesian information criterion; DAPC, discriminate analysis of principle component. (TIF) [file pbio.3001890.s002.tif]

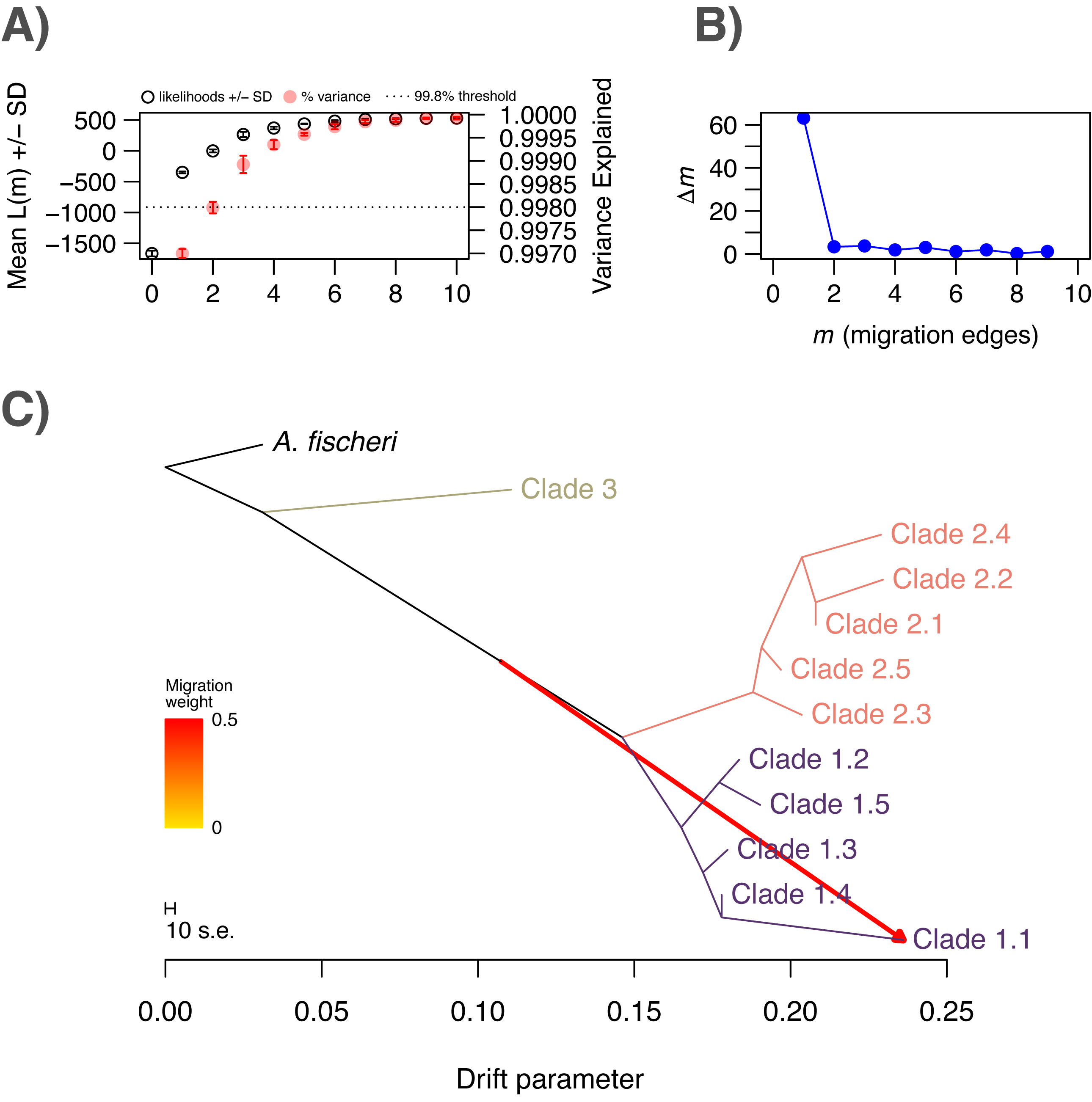

Supplement: S4 Fig — TreeMix was run on a VCF file containing all strains plus A. fischeri as the outgroup with the optimal number of migration edges determined using the program OptM, using 10 independent iterations of m ranging from 1–10. (A) Variance explained across different values of m and (B) delta m spike supporting the optimal migrations at m = 1. (C) TreeMix graph supporting gene flow from Clade 3 to sub-clade 1.1, containing 5 introgressed isolates with majority ancestry in Clade 1 and including the reference strain Af293. The data underlying this figure can be found in DOI: 10.5281/zenodo.5775265. (TIF) [file pbio.3001890.s004.tif]

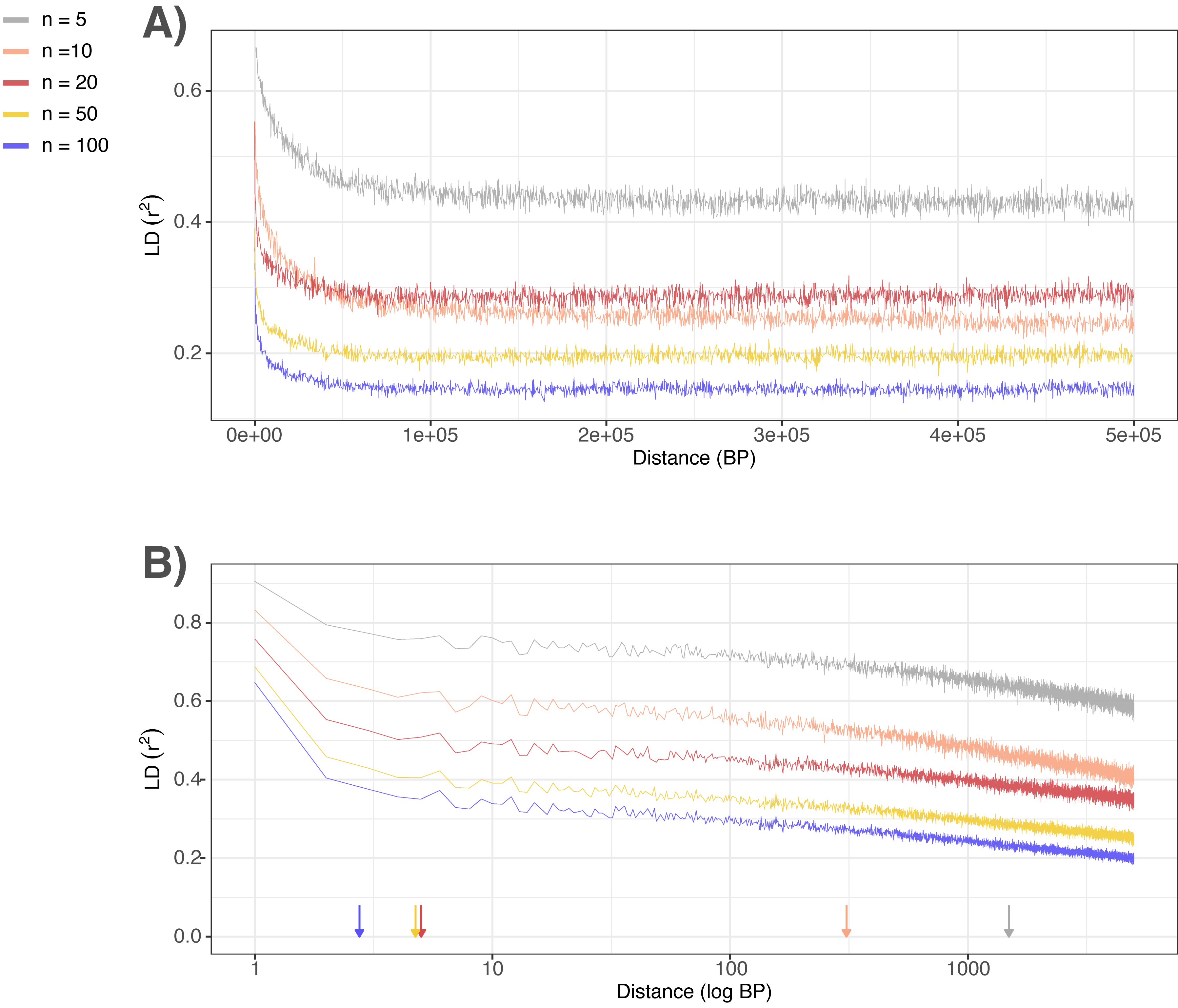

Supplement: S5 Fig — To investigate the influence of sample size on LD decay, we iteratively sampled n = 5, 10, 20, 50, or 100 isolates without regard to population structure (each n averaged over 20 independent iterations) and calculated LD50 in BPs. The influence of sample size on LD estimates showed a strong inverse relationship between sample size and LD decay, with an LD50 of 1,489.55 BP at n = 5, 308.80 BP at n = 10, 5.02 BP at n = 20, 4.7 BP at n = 50, and 2.76 BP at n = 100. (A) Linear–linear plot. (B) Zoomed in log-linear plot, with arrows denoting LD50 at each n. The data underlying this figure can be found in DOI: 10.5281/zenodo.5775265. BP, base pair; LD, linkage disequilibrium. (TIF) [file pbio.3001890.s005.tif]

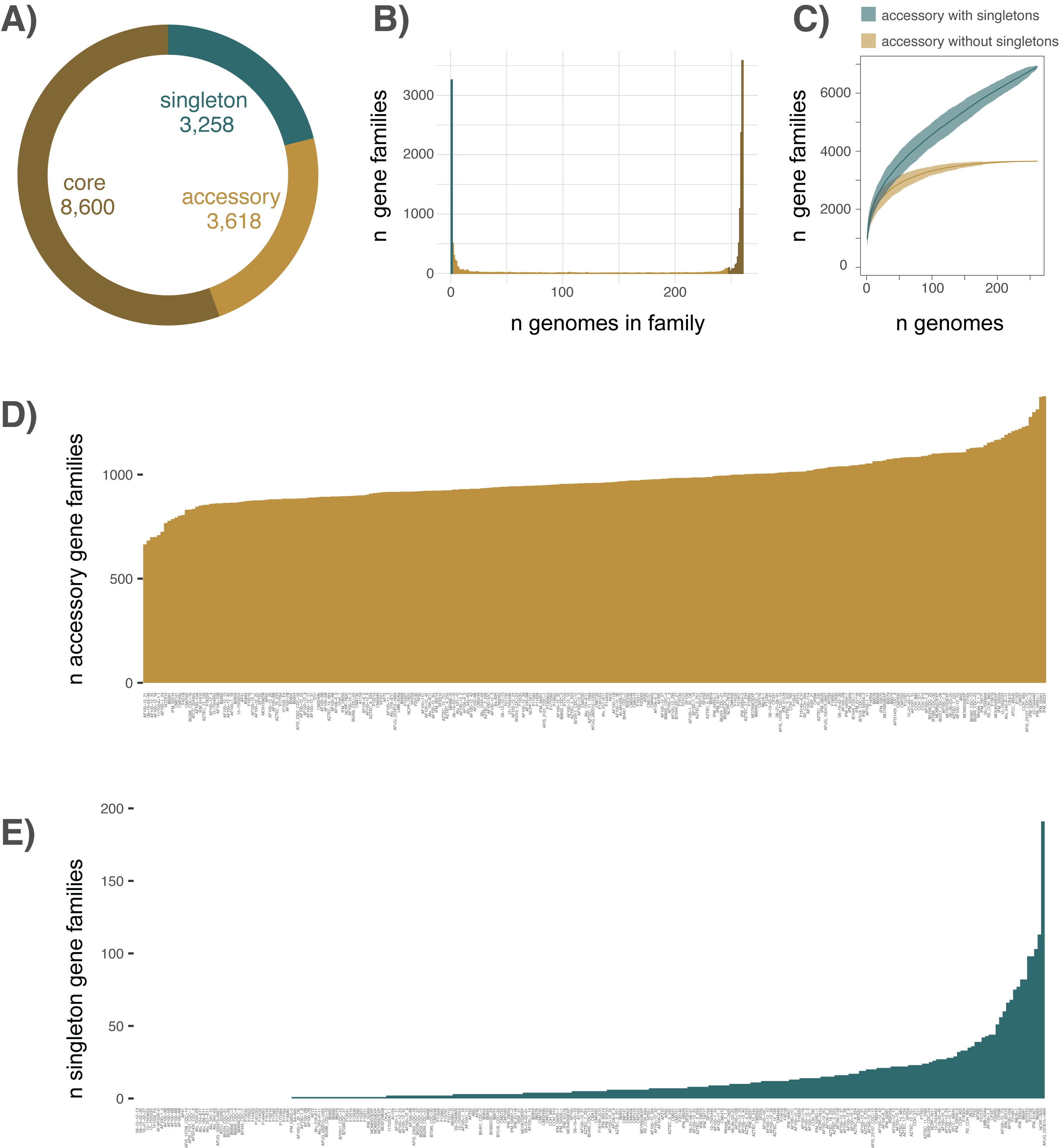

Supplement: S6 Fig — (A) The pan-genome of 260 A. fumigatus strains included 15,476 gene families in total, including 8,600 (55.57%) core genes (present in >95% of strains), 3,618 (13.92%) accessory genes (present in >2 and <248 strains), and 3,258 (21.05%) singletons (present in only 1 isolate). (B) The distribution of the number of genomes represented in each gene family. (C) Gene family accumulation curves, including (green) and excluding (yellow) singletons. (D) The distribution of unique accessory gene families by strain. (E) The distribution of unique singleton gene families by strain. The data underlying this figure can be found in DOI: 10.5281/zenodo.5775265. (TIF) [file pbio.3001890.s006.tif]

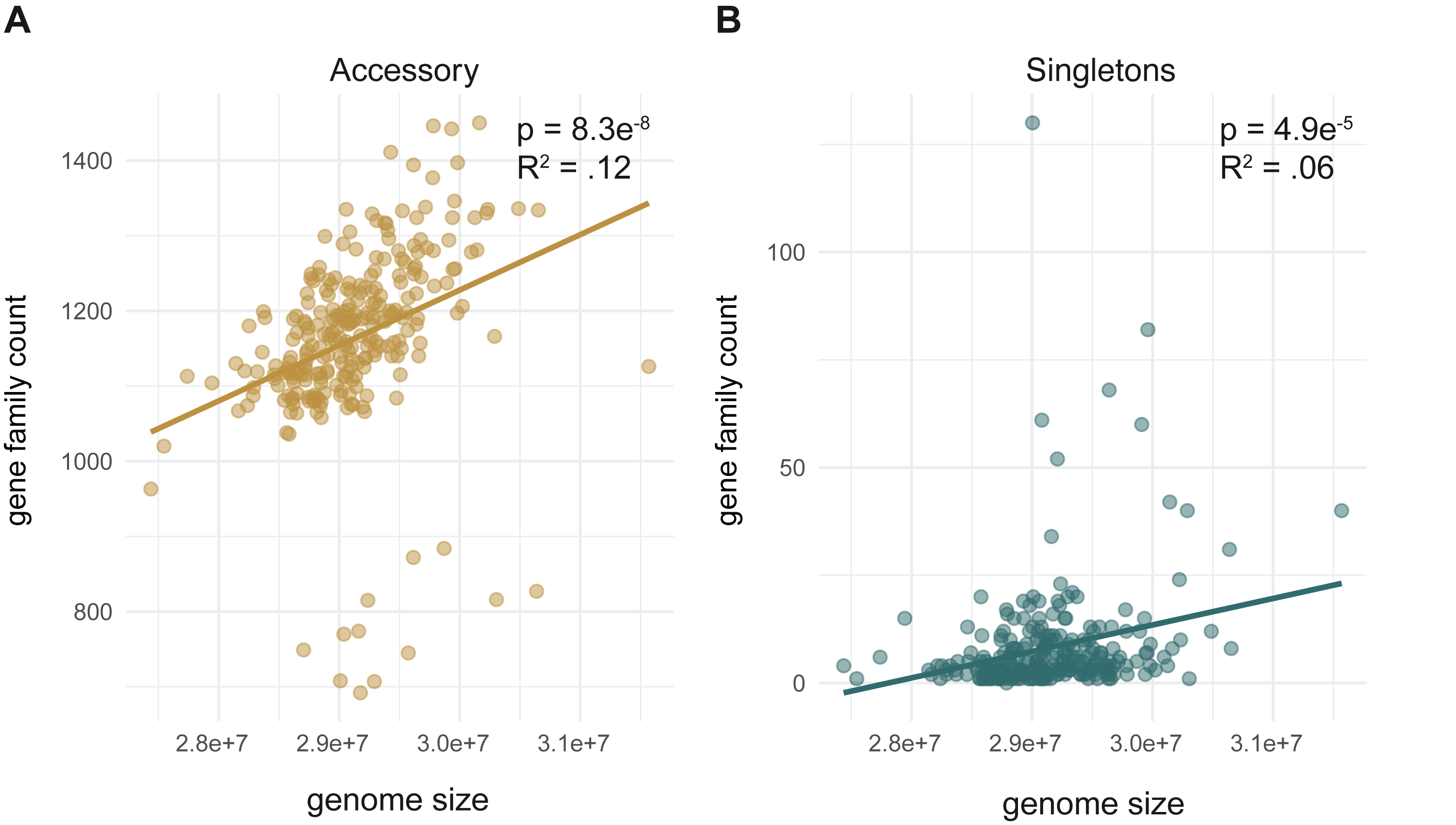

Supplement: S7 Fig — Both (A) accessory gene families and (B) singleton gene families were significantly related to predicted genome size, although this relationship yielded low R2 values, particularly for singleton gene families. The data underling this figure can be found in https://github.com/MycoPunk/Afum_PopPan. (TIF) [file pbio.3001890.s007.tif]

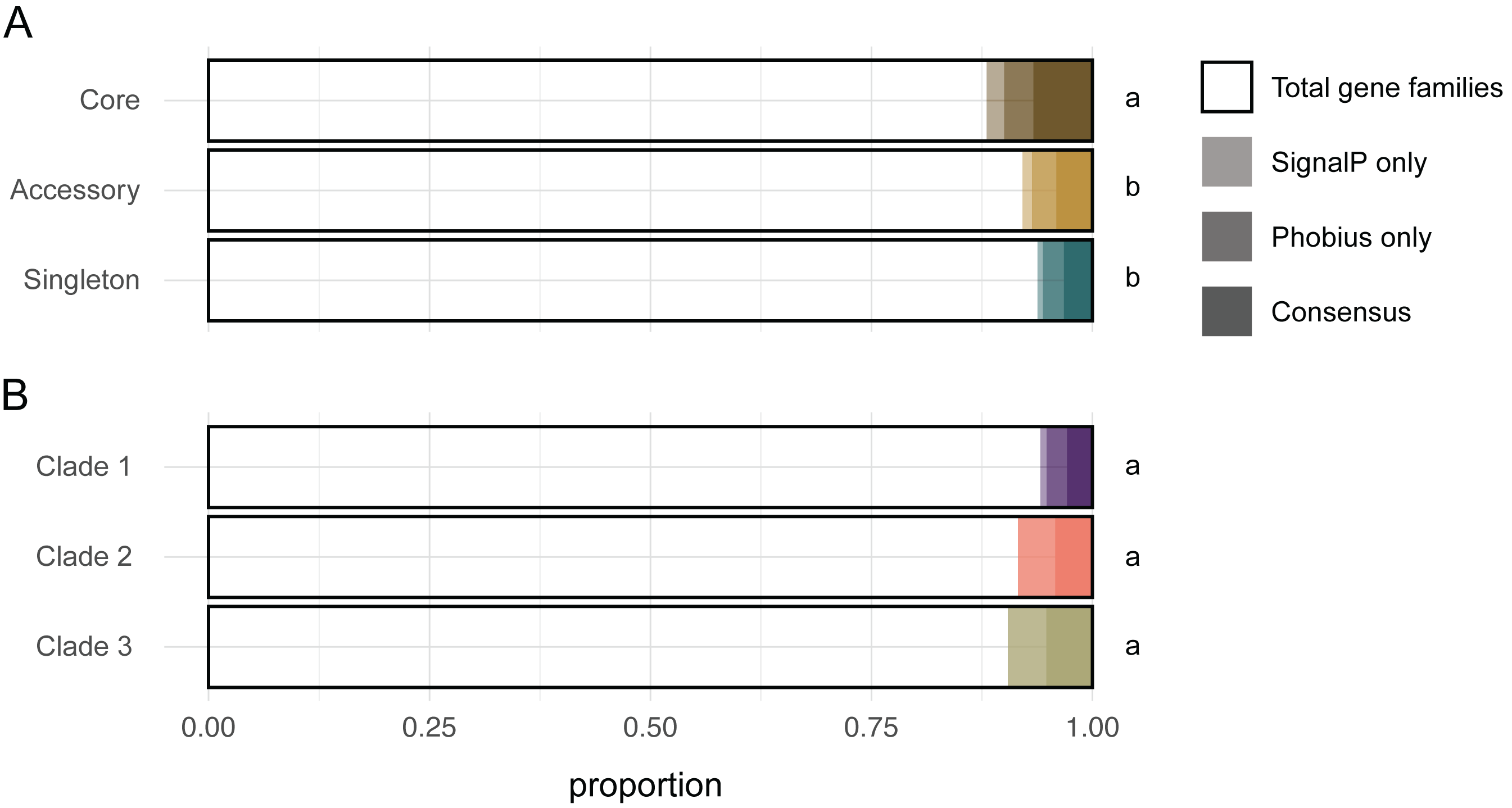

Supplement: S8 Fig — Secreted proteins were predicted using the programs Signal P and Phobius. In most cases, Phobius predicted a slightly greater number of secreted proteins than Signal P. Both Signal P (lightest shading) and Phobius (mid-level shading) contained proteins not annotated by the other program, but most individual proteins called as secreted overlapped between the 2 prediction programs (darkest shading). (A) Proportion of gene families predicted to represent secreted proteins out of all gene families by frequency (core, accessory, and singleton) and (B) by clade for clade-specific gene families. In Clade 3, only 2 categories are depicted as all secreted proteins predicted by Signal P were also predicted by Phobius. Significant differences were assessed using Pairwise proportion tests with Bonferroni adjustment for multiple comparisons at p < 0.05. Letters (lowercase) in common, indicate no significant difference between groups. Significance results were consistent across Signal P, Phobius, and Consensus inferred secreted proteins. The data underlying this figure can be found in DOI: 10.5281/zenodo.5775265. (TIF) [file pbio.3001890.s008.tif]

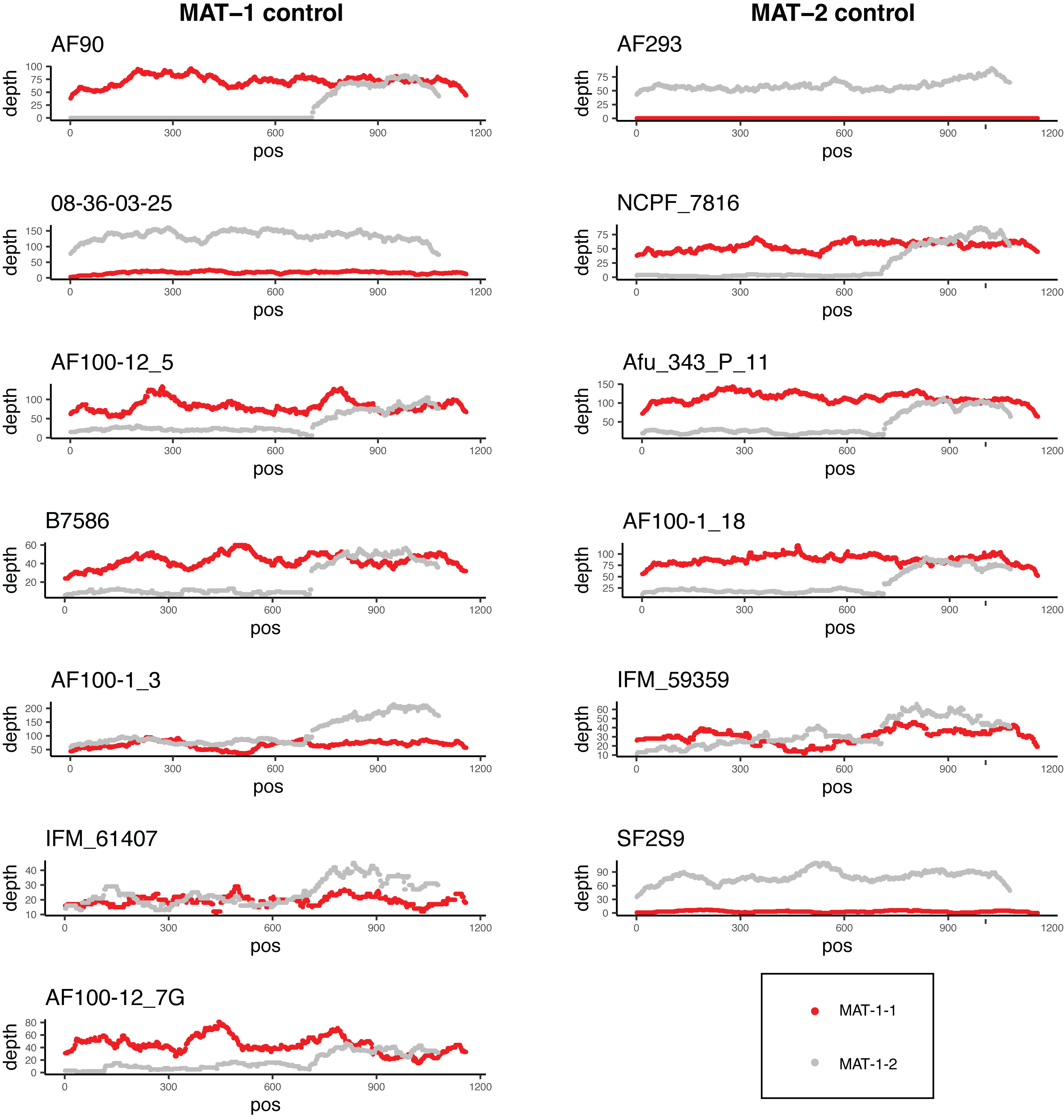

Supplement: S9 Fig — All strains had alignments over the entirety of the MAT region for both idiomorphs, but these alignments different in depth, suggesting different underlying explanations. For MAT1-2 alignments, the trailing approximately 270 BP region that is conserved between MAT1-1 and MAT1-2 is visible here in the control as well as in several of the isolates that mapped to both idiomorphs. While MAT1-2 idiomorph strains have a full alignment over the (approximately 1,078 BP) MAT1-2 reference, MAT1-1 idiomorph strains only align over the approximately 270 BP conserved region of the MAT1-2 reference. The data underlying this figure can be found in DOI: 10.5281/zenodo.5775265. (TIF) [file pbio.3001890.s009.tif]

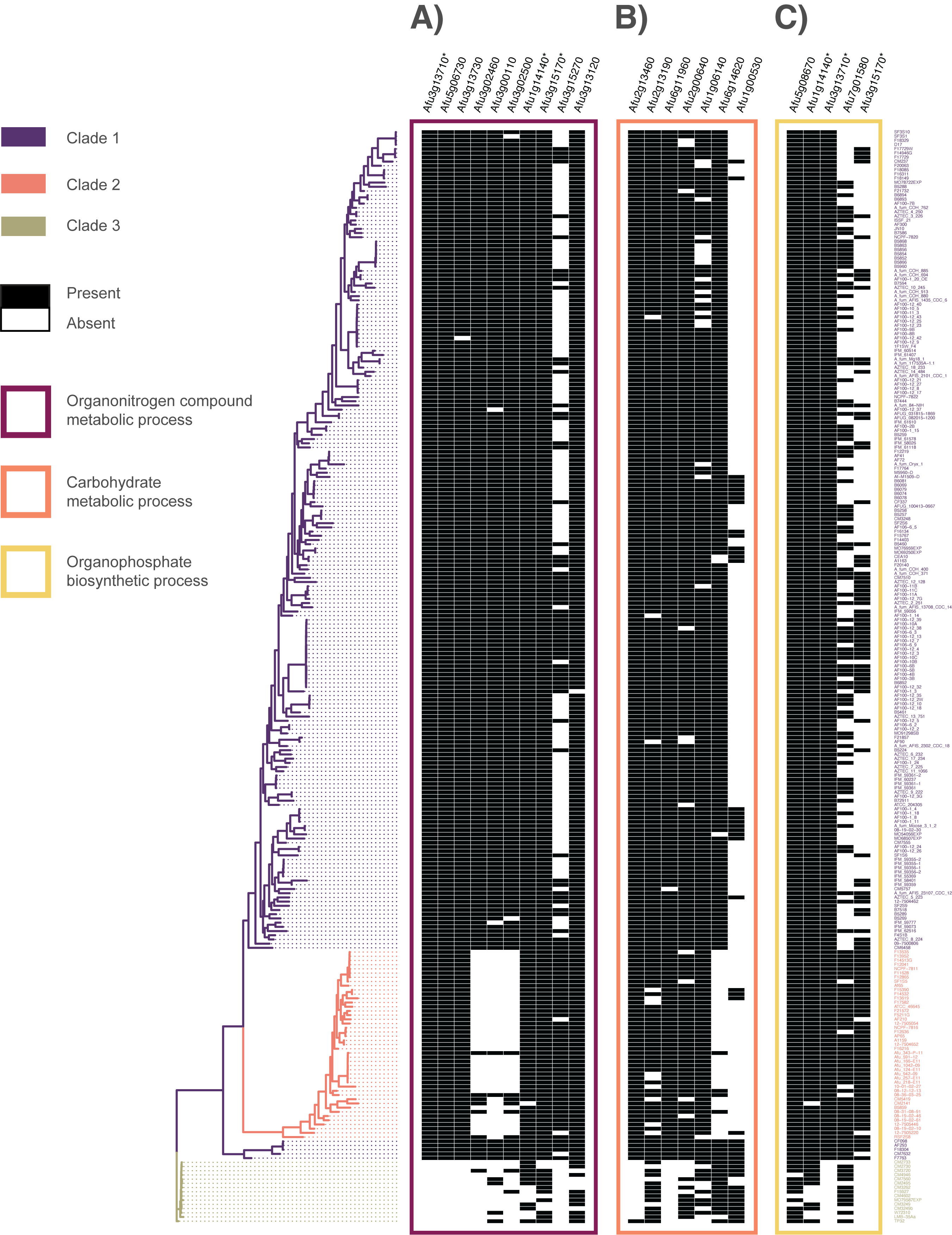

Supplement: S12 Fig — Gene family clusters with significant homology to annotated genes the Af293 reference genome were assigned using BLASTP (e-value < 1e-15). Using these annotated clusters, we identified differential abundance in clusters with Af293 annotations between the 3 clades, for Af293 genes in FungiDB under the GO terms (A) organonitrogen compound metabolic process (GO: 1901564, n = 25 significantly differentially abundant genes; only the first 7 are depicted for visualization), (B) carbohydrate metabolic process (GO: 0005975, 7 significantly differentially abundant genes), and (C) organophosphate biosynthetic process (GO: 0090407, 5 significantly differentially abundant genes). Genes denoted with * are shown grouped with GO: 1901564, but are also annotated in GO: 0090407. The data underlying this figure can be found in DOI: 10.5281/zenodo.5775265. (TIF) [file pbio.3001890.s012.tif]

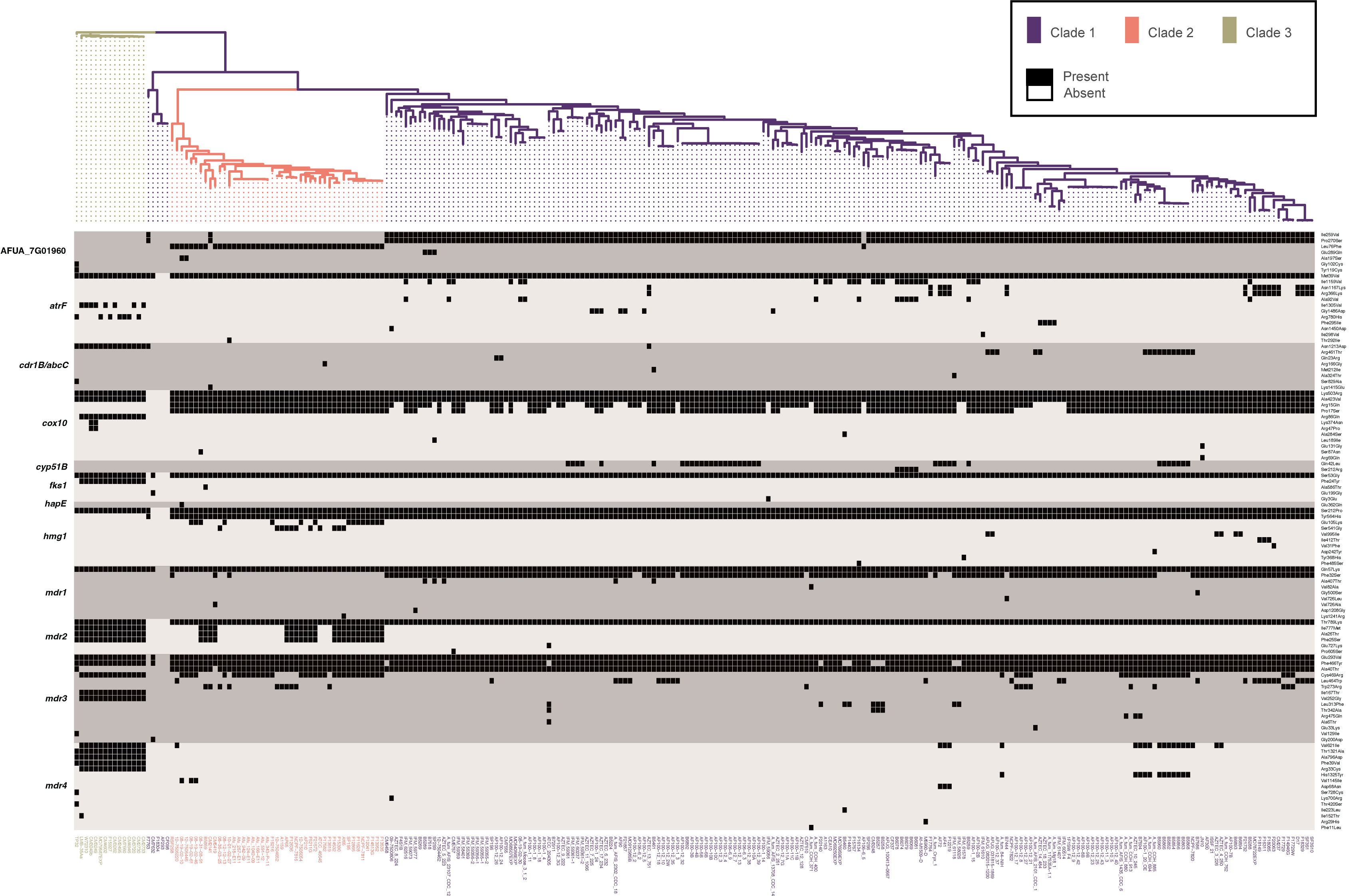

Supplement: S13 Fig — In addition of cyp51A, a database of other genes known to be associated with antifungal drug resistance was assembled (S2 Table), and all isolates were scanned for non-synonymous variants in these genes. Four genes, Cyp51b, cox10, mdr2, and AFUA 7G01960 contained uncharacterized non-synonymous amino acid changes, several of which demonstrated phylogenetic structure. The data underlying this figure can be found in DOI: 10.5281/zenodo.5775265. (TIF) [file pbio.3001890.s013.tif]

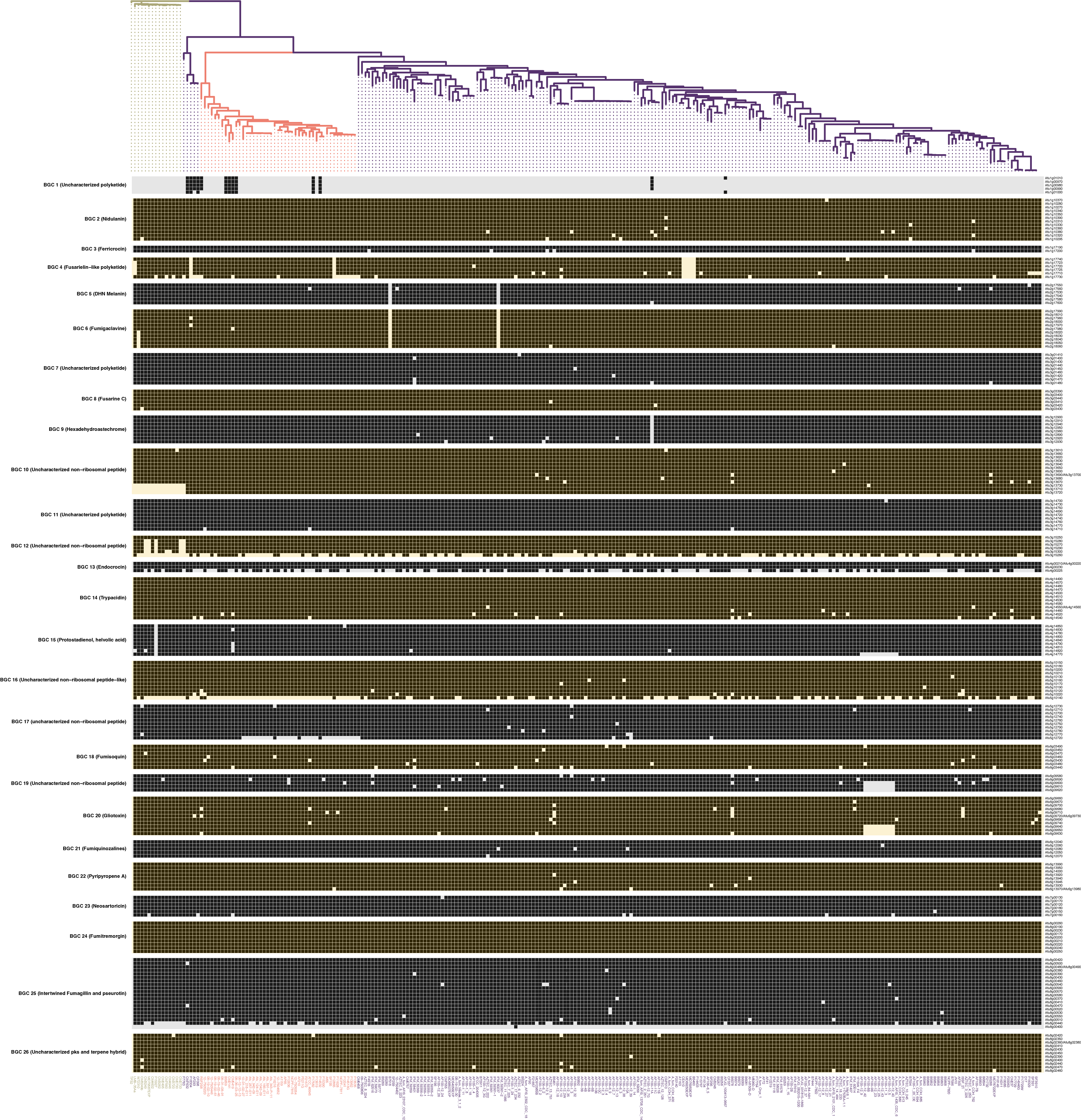

Supplement: S14 Fig — Gene family clusters with significant homology to annotated genes the Af293 reference genome were assigned using BLASTP (e-value < 1e-15) and genes in gene clusters encoding notable A. fumigatus secondary metabolites (as defined in [112]) were mapped onto the phylogeny in R. Out of 230 genes in 26 clusters, 7 were convoluted with one other gene in the cluster, where both genes clustered into the same gene family (in these cases, both genes are listed separated by /). Three genes (Afu1g10275 in cluster 2, Afu7g00140 in cluster 23, and Afu8g00450 in cluster 25) could not be confidently assigned to an Orthofinder gene family and are not displayed. The data underlying this figure can be found in DOI: 10.5281/zenodo.5775265. BGC, biosynthetic gene cluster. (TIF) [file pbio.3001890.s014.tif]

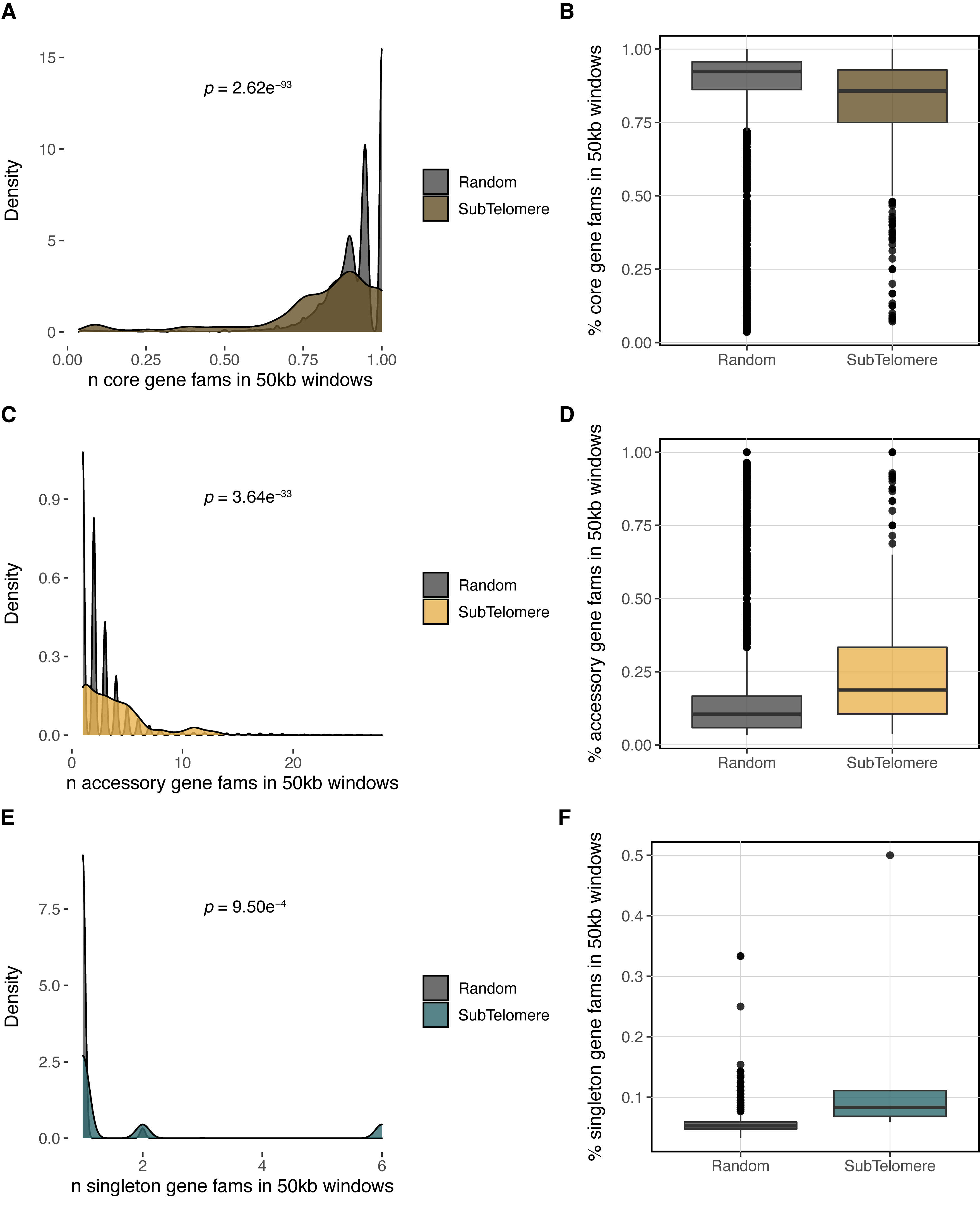

Supplement: S15 Fig — The relative abundance of core, accessory, and singleton gene families was calculated over 50 kb windows for all contigs >50 kb in length and containing a telomeric repeat. Possible enrichment of each abundance category was then tested using a 2-sided Wilcoxon rank sum test implemented in the stats package in R, showing significant depletion of core genes and significant enrichment of singleton and accessory genes within 50 kb of telomere ends. The data underlying this figure can be found in DOI: 10.5281/zenodo.5775265. (TIF) [file pbio.3001890.s015.tif]

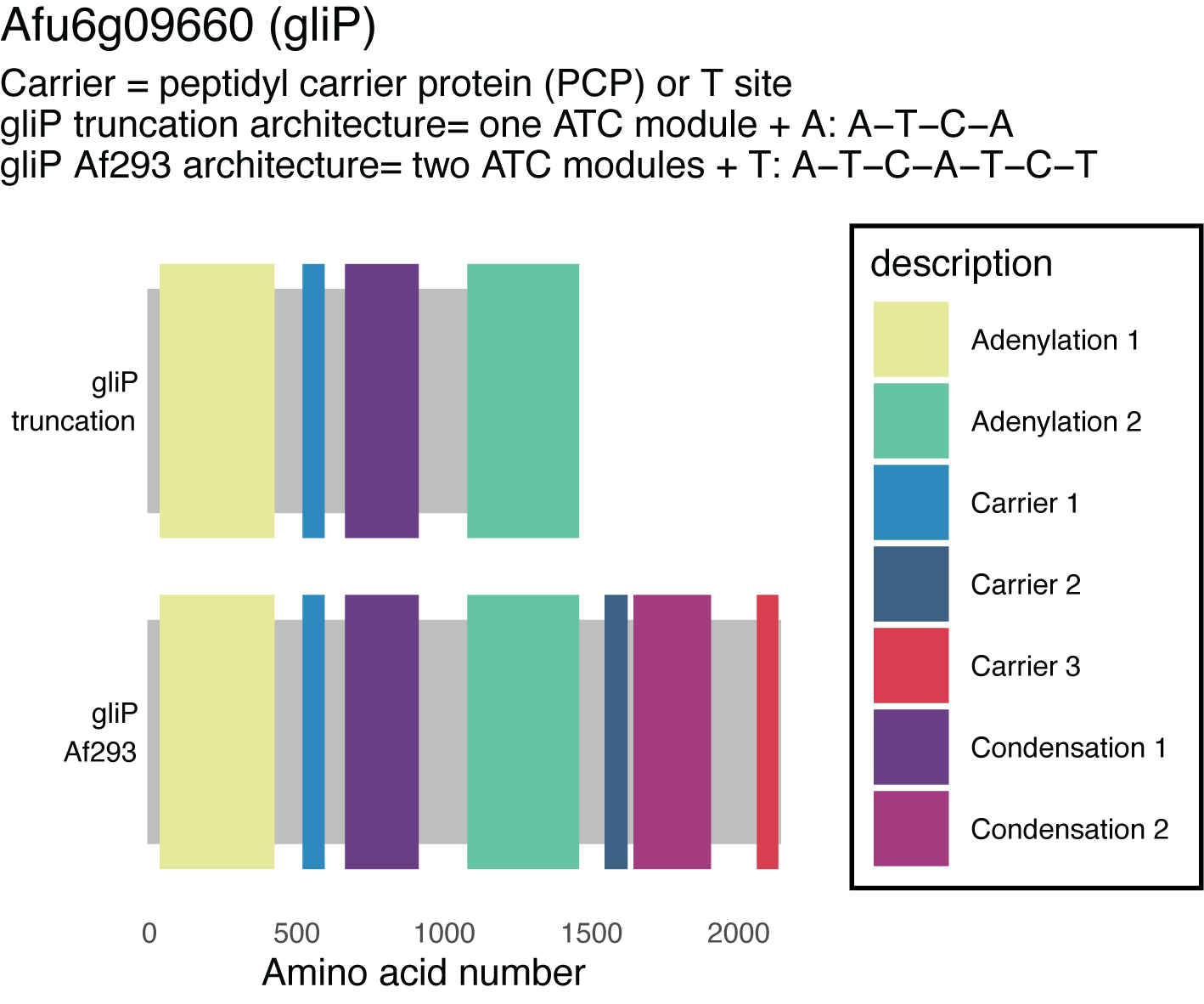

Supplement: S16 Fig — The boundaries of the deletion were the same for all 8 strains and included the partial truncation of gliP, covering 1 A-T-C module. The data underlying this figure can be found in DOI: 10.5281/zenodo.5775265. (TIF) [file pbio.3001890.s016.tif]
